# AP1/Fra1 confers resistance to MAPK cascade inhibition in pancreatic cancer

**DOI:** 10.1007/s00018-022-04638-y

**Published:** 2022-12-19

**Authors:** Christian Schneeweis, Sandra Diersch, Zonera Hassan, Lukas Krauß, Carolin Schneider, Daniele Lucarelli, Chiara Falcomatà, Katja Steiger, Rupert Öllinger, Oliver H. Krämer, Alexander Arlt, Marian Grade, Marc Schmidt-Supprian, Elisabeth Hessmann, Matthias Wirth, Roland Rad, Maximilian Reichert, Dieter Saur, Günter Schneider

**Affiliations:** 1 Medical Clinic and Polyclinic II, Klinikum Rechts Der Isar, Technical University Munich, 81675 Munich, Germany; 2 Institute for Translational Cancer Research and Experimental Cancer Therapy, Technical University Munich, 81675 Munich, Germany; 3 Department of General, Visceral and Pediatric Surgery, University Medical Center Göttingen, 37075 Göttingen, Germany; 4 Comparative Experimental Pathology, Institute of Pathology, School of Medicine, Technical Universität München, 81675 Munich, Germany; 5 German Cancer Research Center (DKFZ), German Cancer Consortium (DKTK), 69120 Heidelberg, Germany; 6 Institute of Molecular Oncology and Functional Genomics, TUM School of Medicine, TU München, 81675 Munich, Germany; 7 Department of Toxicology, University of Mainz Medical Center, 55131 Mainz, Germany; 8 Department for Internal Medicine and Gastroenterology, University Hospital, Klinikum Oldenburg AöR, 26133 Oldenburg, Germany; 9 CCC-N (Comprehensive Cancer Center Lower Saxony), Göttingen, Germany; 10 Institute of Experimental Hematology, School of Medicine, Technical University of Munich, 81675 Munich, Germany; 11 University Medical Center Göttingen Department of Gastroenterology, Gastrointestinal Oncology and Endocrinology, 37075 Göttingen, Germany; 12 Clinical Research Unit 5002, KFO5002, University Medical Center Göttingen, 37075 Göttingen, Germany; 13 Department of Hematology, Oncology and Tumor Immunology, Campus Benjamin Franklin, Charité—Universitätsmedizin Berlin, 12203 Berlin, Germany; 14 Translational Pancreatic Research Cancer Center, Medical Clinic and Polyclinic II, Klinikum Rechts Der Isar, Technical University Munich, 81675 Munich, Germany

**Keywords:** Pancreatic cancer, KRAS, MEK, ERK, AP1, FRA1

## Abstract

Targeting KRAS downstream signaling remains an important therapeutic approach in pancreatic cancer. We used primary pancreatic ductal epithelial cells and mouse models allowing the conditional expression of oncogenic Kras^G12D^, to investigate KRAS signaling integrators. We observed that the AP1 family member FRA1 is tightly linked to the KRAS signal and expressed in pre-malignant lesions and the basal-like subtype of pancreatic cancer. However, genetic-loss-of-function experiments revealed that *FRA1* is dispensable for Kras^G12D^-induced pancreatic cancer development in mice. Using FRA1 gain- and loss-of-function models in an unbiased drug screen, we observed that FRA1 is a modulator of the responsiveness of pancreatic cancer to inhibitors of the RAF–MEK–ERK cascade. Mechanistically, context-dependent FRA1-associated adaptive rewiring of oncogenic ERK signaling was observed and correlated with sensitivity to inhibitors of canonical KRAS signaling. Furthermore, pharmacological-induced degradation of FRA1 synergizes with MEK inhibitors. Our studies establish FRA1 as a part of the molecular machinery controlling sensitivity to MAPK cascade inhibition allowing the development of mechanism-based therapies.

**Supplementary Information:**

The online version contains supplementary material available at 10.1007/s00018-022-04638-y.

## Introduction

Oncogenic KRAS signaling can initiate and drive carcinogenesis in the pancreas [1–3]. The *KRAS* oncogene is mutated in 90–95% of pancreatic ductal adenocarcinomas (PDACs), and copy number gains were recently connected to the aggressive basal-like/mesenchymal subtype of the disease and the metastatic cascade [4–6]. Such insights demonstrate the value of KRAS as a therapeutic target and direct and specific RAS inhibitors are under pre-clinical and clinical development [7].

Despite the development of mutation-specific KRAS inhibitors [7], targeting the canonical RAF-MEK-ERK signaling pathway downstream of KRAS remains a therapeutic option [8]. However, the clinical implementation of inhibitors of the RAF-MEK-ERK signaling was not successful so far [9, 10], which is due to insufficient on-target activity, adaptation, resistance, a small therapeutic window, or tumor heterogeneity [8, 11]. Knowledge of adaptation and resistance mechanisms was the basis for the development of combination therapies, which includes among others, MEK-ERK inhibitors with up-stream interfering approaches like Src homology region 2 (SH2)-containing protein tyrosine phosphatase 2 (SHP2) inhibitors [12, 13], the blockade of autophagy [14, 15], or inhibitors of the PI3K-AKT-MTOR signaling pathway [16–19]. In addition, the notion that blockade of canonical KRAS signaling leads to adaptive reactivation of the signaling pathway resulted in a concept of low-dose vertical inhibition, which includes the combination of a pan-RAF inhibitor together with an ERK inhibitor. This combination impeded ERK reactivation and switched the cellular response from cytostatic to cytotoxic [20].

It is important to consider that such combination therapies, like the combination of a MEK with an AKT inhibitor, failed in the clinic [21]. Therefore, it is necessary for successful translation that potential combination therapies based on a KRAS-signaling backbone are stratified for the responding population. Here, recent observations that the differentiation state along with the epithelial to the mesenchymal continuum is connected to MEKi sensitivity, whereby an undifferentiated mesenchymal PDAC subtype is less MEKi sensitive [22, 23] and that the epithelial-mesenchymal transition transcription factor SLUG can mediate MEKi resistance [24], may allow for stratification of MEKi-based therapies. However, downstream control of MEKi resistance is incompletely understood so far and further insights and markers are necessary to successfully implement such therapies.

To define integrators of oncogenic KRAS signal transduction, we used primary murine pancreatic ductal epithelial cells (PDECs) allowing to switch on the expression of the KRAS oncogene, driven by the endogenous promoter [25]. Focusing on transcription factors, we observed that the AP1 family member FOSL1/FRA1 (FRA1 afterward) is tightly connected to oncogenic KRAS. Although the *FRA1* gene is dispensable for the Kras^G12D^-driven carcinogenesis in the murine pancreas, FRA1 marks a PDAC subtype with characteristics of basal-like, aggressive PDACs. Unbiased drug screening in isogenic gain- and loss-of-function models showed that FRA1 can modulate MEKi and ERKi sensitivity, which was correlated with different MAPK signaling thresholds.

## Material and methods

### Drugs

The cherry-picked library including the inhibitors trametinib, RO5126766, and ulixertinib were obtained from Selleckchem (Houston, TX, USA). The degrader dTAG13 was purchased from Tocris/Bio-Techne GmbH (Wiesbaden, Germany) and Doxycycline from Cayman Chemical (Ann Arbor, MI, USA).

### Mouse lines

Mouse lines and genotyping of these lines were described recently [25]. Genotyping of *LSL-Kras*^*G12D/*+^ [26] and *R26*^*CreERT2*^ [27] was described [25], and the information for *Pdx1-Flp*, *FSF-Kras*^*G12D*^, and *p53*^*Frt*^ line can be found in [28, 29]. The *Fra-1*^*lox*^ [30] and *Ptf1a*^*Cre*^ [31] mice were genotyped using the following primers: *Fra-1*^*lox*^ 5´- G A A A T G G C T C C G T G G G T A A A G G T A -3´, 5´- G A C A G G G T T C A T C T T C A T A G T T C T -3´, 5´- T G T A C C G G A C G C T T G T C A T C T C A T -3´(408 bp (del), 308 bp (wt), 354 bp (lox); *Ptf1a*^*Cre*^ 5´-C C T C G A A G G C G T C G T T G A T G G A C T G C A -3´, 5´-C C A C G G A T C A C T C A C A A A G C G T -3´, 5´- G C C A C C A G C C A G C T A T C A A -3´ (600 bp (wt), 400 bp (mut)). Animals had a mixed *C57Bl/6*;*129S6/SvEv* genetic background. All animal studies were performed in compliance with European guidelines for the care and use of laboratory animals and were approved by the Institutional Animal Care and Use Committees (IACUC) of Technische Universität München and the Regierung von Oberbayern.

### Murine PDAC, PDEC cell lines, murine 3D culture

Low-passaged murine PDAC cell lines were isolated as described [32]. In brief, small fragments (5 mm diameter) of established murine PDACs were washed in sterile PBS, minced into smaller pieces with sterile scalpels, and transferred to 5 mL DMEM (#D5796, Sigma-Aldrich, Taufkirchen, Germany) with 7 mg of Collagenase Type 2 (Worthington Biochemical Corp., Lakewood, NJ, USA) and incubated at 37 °C overnight. The following day, the samples were centrifuged, the medium removed, the cell pellet resuspended in 5 mL DMEM Medium supplemented with 10% fetal bovine serum (FBS) ((#S0615, Biochrom, /Sigma-Aldrich) and 1% Penicillin–Streptomycin (Thermo Fisher Scientific, Darmstadt, Germany), and transferred into cell culture flasks. Cell lines were cultured in DMEM medium (with 10% FCS and 1% Penicillin–Streptomycin) at 37 °C in a CO_2_ incubator. Authenticity of the murine PDAC cell lines was verified using genotyping PCRs. Murine PDECs were isolated and propagated essentially as described [25]. For the 3D culture, 40,000 cells were seeded in growth-factor-reduced (GFR) Matrigel (BD, Heidelberg, Germany) and cultured in complete medium (AdDMEM/F12 medium (Invitrogen/Thermo Fisher Scientific, Darmstadt, Germany) supplemented with: HEPES (Invitrogen), glutamax (Invitrogen), 1% P/S (Invitrogen), B27 (Invitrogen), primocin (1 mg/ml, InvivoGen), *N*-acetyl-l-cysteine (1 mM, Sigma-Aldrich), RSPO1-conditioned medium (10% v/v), noggin (0.1 mg/ml, Peprotech, Hamburg, Germany), gastrin (10 nM, Sigma-Aldrich), fibroblast growth factor 10 (FGF10, 100 ng/ml, Preprotech), nicotinamide (10 mM, Sigma-Aldrich), and A83-01 (0.5 μM, Tocris/Bio-Techne GmbH, Wiesbaden, Germany)). After 1 week the spheroids were passaged and 3 days after passage; they were fixed with Formalin 4% and stained with Alexa Fluor@ 594 Phalloidin (#A12381, Thermo Fisher Scientific) and ToPro3 Iodide (T3605, Thermo Fisher Scientific).

### Mycoplasma contamination test

Cell lines were tested for mycoplasma contamination by a PCR-based method [33]. Briefly, cells were grown in 6-well-plates in 3 mL regular growth medium without Penicillin/Streptomycin for 10 days, after which 2 mL of the medium was collected and centrifuged at 250×*g* for 2 min. The supernatant was taken off and centrifuged again for 10 min at 16,000×*g*. The pellet was resuspended in 50 µL PBS and boiled at 95 °C for 5 min. From each sample, 2 µL was used as template for the Mycoplasma Detection PCR together with 15 µL Red-Taq Premix (Sigma-Aldrich), 2 µL 5`-Forward-Primer dilution mix (10 µmol/L each primer), 2 µL 3`-Reverse-Primer dilution mix (10 µmol/L each primer), and 9 µL H_2_O. Mycoplasma detection primers are based on the protocol by Uphoff and Drexler [33]: 5’-Forward primer mix contained the following 7 forward primers (10 µmol/L each primer): 5’-Primer-1 5`-C G C C T G A G T A G T A C G T T C G C-3`; 5’-Primer-2 5`-C G C C T G A G T A G T A C G T A C G C-3`; 5’-Primer-3 5`-T G C C T G G G T A G T A C A T T C G C-3`; 5’-Primer-4 5`- T G C C T G A G T A G T A C A T T C G C-3`; 5’-Primer-5 5`-C G C C T G A G T A G T A T G C T C G C-3`; 5’-Primer-6 5`-C A C C T G A G T A G T A T G C T C G C-3`; 5’-Primer-7 5`-C G C C T G G G T A G T A C A T T C G C-3`. The 3’-reverse primer mix contained the following 3 reverse primers (10 µmol/L each primer) 3’-Primer-1 5`-G C G G T G T G T A C A A G A C C C G A-3`; 3’-Primer-2 5`-G C G G T G T G T A C A A A A C C C G A-3`; 3’-Primer-3 5`-G C G G T G T G T A C A A A C C C C G A-3`. PCR was subsequently performed in a thermal cycler with the following settings: Initial denaturation at 95 °C for 15 min, followed by 40 PCR cycles (1 min at 94 °C/1 min at 60 °C/1 min at 72 °C) and a final extension step at 72 °C for 10 min. The PCR samples were separated via gel electrophoresis on a 2% agarose gel and visualized with UVsolo TS Imaging System (Biometra, Analytik Jena AG, Jena, Germany). Only cell lines that tested negative were used in further experiments.

### Reconstitution of FRA1

The coding sequence for murine *Fra1* was amplified from the cDNA of a murine PDAC cell line and cloned into pENTR entry vector (kind gift by Eric Campeau & Paul Kaufman; Addgene plasmid # 17398; RRID:Addgene_17398) [34] using the NEBuilder HiFi DNA Assembly Master Mix (NEB, Frankfurt, Germany) according to the manufacturer’s instructions. The Fra1 cassette was then shuttled by Gateway Cloning into the destination vectors pLenti PGK Puro DEST (kind gift by Eric Campeau & Paul Kaufman, Addgene plasmid # 19068; RRID:Addgene_19068) [34], pLEX_305-N-dTAG (gift from James Bradner & Behnam Nabet, Addgene plasmid #91,797; RRID:Addgene_91797) [35], and pInducer20-Blast (gift from Jean Cook (Addgene plasmid # 109,334; RRID:Addgene_109334) [36] by the use of the Gateway® LR Clonase® II enzyme mix (Thermo Fisher Scientific, Darmstadt, Germany) according to the manufacturer’s instructions. All plasmids were sequenced to control correct insertion, and the plasmids were submitted to Addgene (Addgene Plasmid ID 188665, 188742, and 192267).

For lentiviral production, HEK 293-FT cells were transfected with lentiviral packaging Plasmids (pMD2.G (Addgene plasmid # 12259; RRID:Addgene_12259) and psPAX2 (Addgene plasmid # 12260; RRID:Addgene_12260) (both kindly provided by Didier Trono) and the respective lentiviral plasmids (pLenti-FRA1, pLEX_305-N-dTAG-FRA1, or pInducer-FRA1) using TransIT®-LT1 Transfection Reagent (Mirus Bio LLC, Madison, WI, USA). Lentiviral supernatant was collected, passed through a 0.2-µm filter, and subsequently used for transduction of PDAC cell lines in the presence of 8 µg/ml polybrene (Sigma-Aldrich). Transduced cell lines were selected with 3 µg/mL Puromycin or 10 µg/mL Blasticidin 48 upon transduction for 3–5 days or 7-10 days respectively.

For generation of the dual-recombinase SDF675 dTAG-FRA1 cell line, SDF675 cells *(Pdx1-Flp;FSF-Kras*^*G12D/*+^*, p53*^*frt/*+^*; Fra1*^*lox/lox*^*)* were first transduced with a pInducer20-iCre construct, allowing for the doxycycline-inducible expression of iCre-recombinase. After selection with geneticin (500 µg/mL), cells were transduced with the second construct pLEX_305-N-dTAG-FRA1 as described above.

### Drug screen

The cherry-picked drug library consisting of 99 inhibitors targeting various relevant cancer pathways was purchased from Selleckchem. The two SUMO inhibitors ML-93 and TAK-981 were from Millennium Pharmaceuticals/Takeda (Cambridge, Massachusetts, USA) and were later added to the library. The APE1/Ref-1 redox-specific inhibitor APX2009 [37] was a kind gift of Mark R. Kelley (Indiana University, Indianapolis, IN, USA). With the exception of A-1210477 (2 mM) and APX2009 (100 mM), the starting maximum stock concentration of all drugs was 10 mM. Serial seven-point threefold dilutions of the drugs were prepared in DMSO, pipetted into 384-well plates, and stored at − 80 °C. For the drug screen, the two *Fra1*-deficient KCF cell lines SDF287 and SDF716—transduced either with the control vector pLenti RFP or reconstituted with pLenti FRA1—were seeded out in white 96-well plates at a density of 1000 cells per well in 100 µL DMEM growth medium. The following day, the drug library was transferred at a 1:1000 dilution (0.1 µL/well) with a 96-pin replicator pin tool (V&P Scientific, San Diego, CA, USA) from the drug plates to the cells. Each drug was analyzed in technical duplicates. After each transfer step, the pins were cleaned in DMSO/EtOH (1:1), dried on blot paper for 15 s, and then cleaned in isopropanol twice. Cell viability was measured with CellTiter-Glo reagent (Promega, Walldorf, Germany) after 72 h of treatment. Therefore, the plates were adjusted to room temperature for approximately 30 min, and then, 25 µL of CellTiter-Glo was added to each well. After gentle shaking and 15 min of incubation, the luminescence was measured on a FLUOstar OPTIMA microplate reader (BMG Labtech, Ortenberg, Germany). Area under the curve (AUC) and half-maximal growth inhibitory concentration (GI_50_) values from the results were calculated with the RStudio software tool using a script based on the GRmetrics methodology [38].

### MTT cell growth and viability assays

Thiazolyl Blue Tetrazolium Bromide (MTT) viability assays were used to determine cell growth as well as viability upon inhibitor treatment. For inhibitor treatment, 1000 cells per well were seeded in 100 µL growth medium in 96-well plates. Cell lines harboring the dTAG-fusion construct were seeded out in medium containing 1 µM dTAG13 (or equal volume of DMSO for controls) to induce degradation of the dTAG fusion chimeras, while cell lines with the pInducer-FRA1 construct were seeded in the presence or absence of 100 ng/mL doxycycline. On the following day, cells were treated in technical triplicates with 7-point threefold serial dilutions of the respective drugs or DMSO as control. After 72 h of drug treatment, viability of the cells was measured by Thiazolyl Blue Tetrazolium Bromide (MTT) (Sigma-Aldrich). Briefly, 10 µL MTT solution (5 mg/mL in PBS) was added to the cells and incubated for 4 h in a CO_2_ incubator at 37 °C. Afterward, the medium was discarded and the formed formazan crystals dissolved in 200 µL of ethanol/DMSO (1:1). After 15 min of incubation on a shaker, absorbance (595 nm) was measured in a microplate reader (Multiskan™ FC Microplate Photometer, Thermo Fisher Scientific, Darmstadt, Germany). Absorbance values were normalized to the DMSO treated controls and are displayed as relative viability. For quantification of cell growth, 1,000 cells per well were seeded in triplicates in 100 µL growth medium. Cell lines harboring the dTAG degrader construct were additionally treated one day after seeding with 1 µM dTAG13 (or DMSO as control) to induce degradation of the dTAG-fusion proteins, while cell lines with the pInducer-FRA1 construct were treated with 100 ng/mL doxycycline to induce expression of FRA1. Viability was measured by MTT as described above on days 1, 2, 3, and 4 after seeding. Absorbance values were normalized to measurements on day 1 and are represented as relative growth.

### Clonogenic assay

For determining basal clonogenic growth of SDF287 and SDF716 RFP and FRA1 cell lines, 1,000 cells per well were seeded in technical triplicates with 2 mL growth medium in 6-well plates. After culturing the cells for approximately 10 days until visible colonies had formed, the medium was removed and the cells stained with 0.2% (weight per volume, w/v) crystal violet (Sigma-Aldrich) solution (in dH_2_O with 2% (v/v) ethanol) for 30 min on a shaker at room temperature. The stained plates were washed 3 times with H_2_O, dried overnight and scanned on a flatbed scanner (Seiko Epson, Suwa, Nagano, Japan). To solubilize the crystal violet stain for subsequent quantification, 1% (w/v) sodium dodecyl sulfate (SDS) (Serva Electrophoresis GmbH, Heidelberg, Germany) in H_2_O was added to each well and the plates were agitated on an orbital shaker overnight at room temperature. Absorbance of the dissolved crystal violet stain was measured at 595 nm on a CLARIOstar microplate reader (BMG Labtech GmbH). For inhibitor treatment, 1000 cells per well were seeded in 500 µL growth medium in 24-well plates. The SDF675 dTAG-FRA1 cells were seeded in medium additionally containing 1 µM dTAG13 (or an equal volume of DMSO as control) to induce degradation of the dTAG-FRA1 fusion protein. On the following day, the indicated serial dilutions of the inhibitor or DMSO as control were added to the cells. When the cells in the DMSO-treated control wells reached near confluence after approximately one week, the cells were stained with crystal violet and quantified as mentioned above.

### Non-radioactive electrophoretic mobility shift assay

5 μl (20 pmol/μl) of each oligonucleotide (AP-1 s 5´-DY682- C G C T T G A T G A C T C A G C C G G A A-3`, AP-1as 5´-DY682- C G C T T G A T G A C T C A G C C G G A A—3`) was annealed, and the double-stranded oligonucleotide was diluted 1:200 in ddH_2_O. EMSAs were performed using 2 μg of whole cell extract in a 10 μl binding reaction containing 10 mM Tris, 50 mM NaCl, 4 mM DTT, 0.5% Tween-20, and 1 μl of the double-stranded oligonucleotide. After a binding reaction of 20 min, orange G gel loading dye (New England Biolabs, Ontario, Canada) was added and complexes were resolved on a 5% native polyacrylamide gel in 1 × TGE at 10 V/cm for 30 min at room temperature. Complexes were detected by the Odyssey Infrared Imaging System (Licor, Bad Homburg, Germany).

### Cell lysis and western blotting

Whole cell protein lysates were prepared in ice-cold immunoprecipitation buffer (50 mM HEPES, 150 mM NaCl, 1 mM EDTA, 0.5% NP-40, 10% glycerol) or RIPA buffer [150 mmol/L NaCl, Triton-X 100 1% (v/v), sodium deoxycholate 1% (w/v), SDS 0.1% (w/v), 50 mmol/L Tris–HCl, 2 mmol/L EDTA; pH 7.5] supplemented with protease and phosphatase inhibitors (cOmplete™, Mini, EDTA-free Protease Inhibitor Cocktail, Roche; Phosphatase-Inhibitor-Mix I, Serva, Heidelberg, Germany). Lysates were normalized for protein concentration, heated at 95 °C for 5 min in protein loading buffer (45.6 mM Tris–HCl pH 6.8, 2% SDS, 10% glycerol, 1% β-mercaptoethanol, 0.01% bromophenol blue), and loaded onto 10% SDS–polyacrylamide gels. Proteins were transferred to Immobilon-FL (Merck-Millipore, Darmstadt, Germany) or nitrocellulose (GE Healthcare Life Sciences, Freiburg, Germany) membranes by tank/wet electroblotting. Membranes were blocked in PBS supplemented with 5% skim milk and 0.1% Tween and incubated at 4 °C overnight with the following antibodies: pan-ERK (Cell Signaling Technology, Frankfurt, Germany Cat# 4696, RRID:AB_390780); Phospho-p44/42 MAPK (Erk1/2) (Thr202/Tyr204) (Cell Signaling Technology Cat# 4370, RRID:AB_2315112); Phospho-Akt (Ser473) (Cell Signaling Technology Cat# 4060, RRID:AB_2315049); Akt antibody (Cell Signaling Technology Cat# 9272, RRID:AB_329827); Phospho-FRA1 (Ser265) (Cell Signaling Technology Cat# 3880, RRID:AB_2106922); Fra1 (N-17) (Santa Cruz Biotechnology Cat# sc-183, RRID:AB_2106928); HA-Tag (C29F4) (Cell Signaling Technology Cat# 3724, RRID:AB_1549585); GFP antibody (Thermo Fisher Scientific Cat# A-11122, RRID:AB_221569); HSP 90alpha/beta (F-8) (Santa Cruz Biotechnology Cat# sc-13119, RRID:AB_675659), β-actin (Sigma-Aldrich Cat# A5316, RRID:AB_476743), and α-tubulin (Sigma-Aldrich Cat# T6199, RRID:AB_477583). All antibodies were diluted in 5% skim milk in PBS with 0.1% Tween. Fra1 (N-17) was used at a concentration of 1:500. The GFP antibody as well as all primary antibodies from Cell Signaling Technology was used at a 1:1000 dilution. The loading controls actin, tubulin, and HSP90 were used at 1:5000–1:10,000 dilutions. After overnight incubation with primary antibody, membranes were washed three times with PBS-Tween, incubated with anti-mouse or anti-rabbit secondary antibodies conjugated with DyLight™ 680 (Cell Signaling Technology, anti-mouse: Cat# 5470, RRID:AB_10696895 / anti-rabbit: Cat# 5366, RRID:AB_10693812) or DyLight™ 800 (Cell Signaling Technology, anti-mouse: Cat# 5257, RRID:AB_10693543/anti-rabbit: Cat# 5151, RRID:AB_10697505) (all used at 1:15,000 dilution in 5% skim milk in PBS-Tween) for 1 h at room temperature, and imaged with the Odyssey Infrared Imaging System (Licor, Bad Homburg, Germany). Licor Image Studio Lite (Image Studio Lite, RRID:SCR_013715) was used for quantification of phospho- and total-ERK (pan-ERK) and phsopho-AKT and pan-AKT levels.

### Histochemistry, immunohistochemistry, fluorescence microscopy

For histopathology, pancreatic tissues were fixed in 4% formaldehyde (Carl Roth, Karlsruhe, Germany), embedded in paraffin, and sectioned (3 μm thick). Haematoxylin and eosin staining was described [32, 39]. For immunodetection, tissue sections were dewaxed, rehydrated, and placed in a microwave (10 min., 600 W) to recover antigens before incubation with the primary antibody (murine Fra1: 1:100, N-17 Santa Cruz Biotechnology (sc-183); human Fra1: 1:50, C-12 Santa Cruz Biotechnology (sc-28310); α-SMA: 1:100, ab5694, abcam, Cambridge, UK). Biotin-conjugated secondary antibodies were purchased from Vector Laboratories, Peterborough, UK. Peroxidase linked to streptavidin was used with 3,3’-diaminobenzidine tetrahydrochloride (Vectastain® elite ABC Kit and DAB Peroxidase Substrat Kit, Vector Laboratories) as chromogen for detection. Counterstaining was performed with hematoxylin. The human pancreas cancer tissue array including 20 PDACs (Cat No: PAC481) were purchased from Pantomics (San Francisco, CA, USA). FRA1 staining intensity 1 (low staining intensity), 2 (moderate staining intensity), and 3 (strong staining intensity) was determined by two independent investigators (D.S. and G.S.). Cryosections were used for immunoflourescence microscopy. Tissues were fixed in 4% buffered formalin at 4 °C for 2 h followed by dehydration in sucrose solution (15% sucrose, 4 h, 30% sucrose over night, 4 °C). Dehydrated tissues were embedded in Tissue-Tek (Sakura, Torrance, CA) and snap-frozen liquid nitrogen. 10-μm-thick frozen sections were postfixed for 1 min in 4% buffered formalin and washed twice in PBS. Sections were blocked in PBS containing 3% (w/v) bovine serum albumin (BSA), 1% (w/v) Saponin and 1% (v/v) Triton X-100 for 1 h. Nuclei were stained with TOPRO-3-iodide (1:1.000, Invitrogen) and actin filaments with Alexa Fluor 594–linked phalloidin (1:250, Invitrogen) for 2 h at RT. Sections were rinsed three times with PBS, and slides were mounted in Vectashield Mounting Medium (Vector Laboratories, Burlingame, CA). Images were captured and analyzed using AxioVision 4.3 software (Carl Zeiss, Jena, Germany). Quantification and grading of mouse ADM and PanIN lesions were performed on three sections per mouse and depicted as lesions per low power field (200 × magnification). Confocal microscopy was performed using Leica TCS SP8. Diameter measurement of the spheroids was done using z-stack pictures of the spheroids (Imaris Software, Bitplane).

### Quantitative reverse-transcriptase PCR

Total RNA was isolated using the RNeasy Mini-Kit (Qiagen) and cDNA synthesized with TaqMan® Reverse Transcription Reagents (Thermo Fisher Scientific). Quantitative analysis by qPCR was performed as previously described (StepOnePlus, PE Applied Biosystems) [40]. Primer sequences (forward/reverse): *cypA* 5´-A T G G T C A A C C C C A C C G T G T-3´ / 5´-T T C T G C T G T C T T T G G A A C T T T G T C-3´; *Fra1 (E1-E2)* 5´- C G C A A G C T C A G G C A C A G A-3´ / 5´-A A T G A G G C T G C A C C A T C C A-3´; *Fra1* (E3-E4) 5´-C G G C C A G G A G T C A T A C G A G-3´ / 5´-C T T C C A G C A C C A G C T C A A G-3’; *p19*^*ARF*^ (p19-TM-for) 5´-T C G C A G G T T C T T G G T C A C T GT-3´ / *p19*^*ARF*^ (p19-TM-rev) 5´-G A A C T T C A C C A A G A A A A C C C T C T C T-3´.

### RNA-seq, sc-RNA-seq, GSEA, STRING analysis, CRISPR-drop out data, and transcriptome profiles

RNA for the RNA-seq of the *Fra1*-deficient KCF cell lines SDF287, SDF419, and SDF716—transduced with control vector pLenti RFP, reconstituted with pLenti FRA1, or pInducer-FRA1—was isolated using the Maxwell® 16 LEV simplyRNA Tissue Kit and instrument (Promega) following the manufacturer`s instructions (from *n* = 4 biological replicates per sample). RNA sequencing was performed at the Sequencing Core Unit at the TranslaTUM, Technical University Munich (TUM). Library preparation for bulk sequencing of poly(A)-RNA was done as described previously [41]. Barcoded cDNA of each sample was generated with a Maxima RT polymerase (Thermo Fisher Scientific) using an oligo-dT primer containing barcodes, unique molecular identifiers (UMIs), and an adaptor. Ends of the cDNAs were extended by a template switch oligo (TSO), and full-length cDNA was amplified with primers binding to the TSO-site and the adaptor. NEB UltraII FS kit (New England Biolabs, Frankfurt, Germany) was used to fragment cDNA. After end repair and A-tailing, a TruSeq adapter was ligated and 3’-end-fragments were finally amplified using primers with Illumina P5 and P7 overhangs. In comparison with Parekh et al. [41], the P5 and P7 sites were exchanged to allow sequencing of the cDNA in read1, and barcodes and UMIs in read2 to achieve a better cluster recognition. The library was sequenced on a NextSeq 500 (Illumina, San Diego, CA, USA) with 67 cycles for the cDNA in read1 and 16 cycles for the barcodes and UMIs in read2. Data were processed using the published Drop-seq pipeline (v1.0) to generate sample- and gene-wise UMI tables [42]. Reference genome (GRCm38) was used for alignment. Transcript and gene definitions were used according to the GENCODE Version M25. RNAseq analysis was performed with R-Studio (R version 4.0.2 (2020-06-22), open-source license) and DEseq2 (Version 1.33.5). Genes with sum (read counts) < *n* (*n* = number of Samples) were removed, and remaining counts were normalized and transformed using regularized log transformation (rlog) implemented in the DEseq2 package.

The rlog normalized matrix was used to perform a gene set enrichment analysis using the GSEA tool from the Broad Institute (Gene Set Enrichment Analysis, RRID:SCR_003199, http://www.broadinstitute.org/gsea/) [43]. To identify common pathways regulated by FRA1, *Fra1*-deficient cell lines (SDF287 RFP + SDF716 RFP) were compared with FRA1 reconstituted cell lines (SDF287 FRA1 + SDF716 FRA1) using the Gene Ontology_biological process (GO_BP) terms. To find pathways activated after the expression of Kras^G12D^ in PDECs, we analyzed recently published expression profiles (EMBL-EBI ArrayExpress Accession number: E-MTAB-2592). Microarray data of untreated and 4-OHT treated PDECs were collapsed to max. probe using the GSEA app 4.1.0. Collapsed data were analyzed with a GSEA (GSEA app 4.2.3) using default settings (permutation type: gene_set) using the MSigDB (c3.tft.v7.5.1) transcription factor signatures. The signatures with a FDR *q* < 0.05 were depicted.

The PAAD dataset of the TCGA (RNA-seq V2) was downloaded via the cBioPortal platform (http://www.cbioportal.org/) (12/2018). Normal tissues, non-PDAC`s, or samples with low cellularity were excluded according to Peran et al. [44]. Normalized human PDAC RNA-seq data (ICGC) were obtained from the supplementary information of Bailey and colleagues [45], and acinar cell carcinomas and the intraductal papillary mucinous neoplasms (IPMNs) were excluded as described [46]. Normalized RNA-seq data of primary human PDAC cell lines (PDCL) were from the supplementary material of Brunton et al. [47], and mRNA expression profiles of primary murine PDAC (KC) cells were recently described [6]. mRNA expression profiles of PDAC annotated to basal-like A, basal-like B, classical A, and classical B were described [4]. GSEA of the TCGA, ICGC, primary patient-derived cells, and murine PDAC cells was performed with the GeneTrail 3.0 web tool [48].

Single-cell (sc) RNA-seq analysis was performed using the SCANPY toolkit [49]. Data for scRNA-seq analysis of FRA1 in human PDAC were retrieved from [50], which aims to provide a reference single-cell atlas for PDAC by combining scRNA-seq studies. Downloaded data were already preprocessed, quality-controlled, count-normalized, and the cell clusters were already annotated. The data encompassed 72 samples with a total of 136,163 cells. The average counts per cell were 8507 and an average detection of 2244 genes per cell. Log10 transformation was performed on the normalized counts. PCA Analysis demonstrated that the first 15 components carried the majority of the variance. A batch correction was performed based on the patient (sample) using BBKNN and the 15 PCA components according to [51] and UMAP according to [52]. Cell type/tumor-subtype signature scores were directly taken from the publication [50] to compute Pearson Correlation between *FRA1* expression and cell type/subtype scores.

The STRING analysis was performed via the STRING web tool (version 11.5) (https://string-db.org/cgi/) [53]. The multiple protein mode with the drug targets as input was used, a highest confidence interaction score (0.9) applied, and interactors were restricted to the input. Furthermore, CRISPR/Cas drop-out gene effects for the AP1 family were accessed via the DepMap portal (https://depmap.org) [54].

### Statistical methods

Unless otherwise indicated, all data were determined from at least three independent experiments and are presented as mean ± standard deviation (SD). A two-sided Student`s *t*-test or one-way/two-way ANOVA was used to investigate statistical significance. *P*-values were calculated with GraphPad Prism (GraphPad Software, San Diego, CA, USA) and corrected for multiple testing.

## Results

### *Kras*^*G12D*^* induces FRA1 in PDECs*

**Fig. 1:**
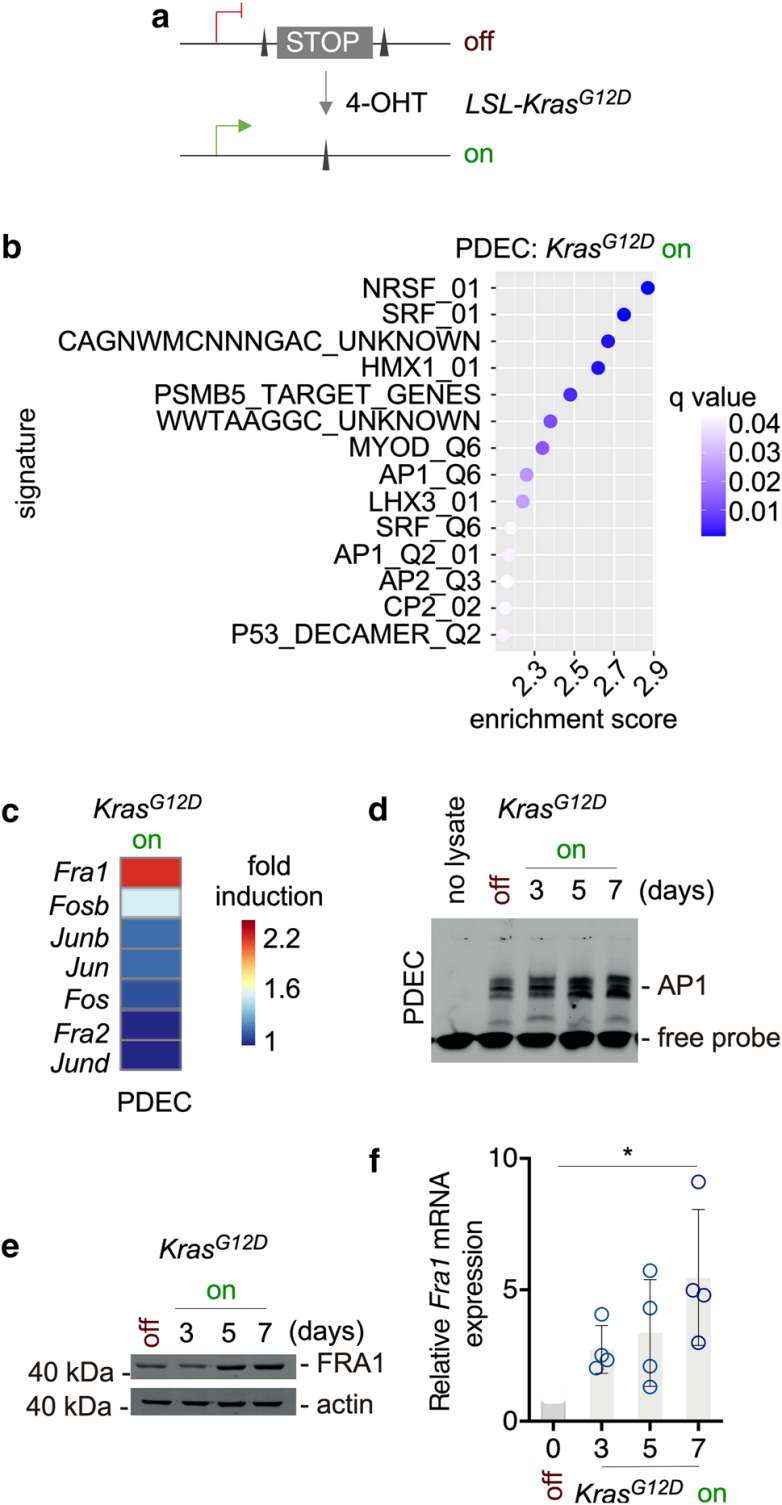
AP-1 transcription factors are linked to oncogenic KRAS in murine primary pancreatic epithelial cells (PDEC). **a** Genetic strategy to activate oncogenic *Kras*^*G12D*^ expression in PDECs isolated from *R26*^*CreERT2*^*;LSL-Kras*^*G12D/*+^ mice. Treatment with 4-OHT induces Cre-mediated recombination of the STOP cassette to activate the expression of oncogenic Kras^G12D^. **b** Transcription factor gene sets upregulated upon activation of oncogenic *Kras*^*G12D*^. PDECs from *R26*^*CreERT2*^*;LSL-Kras*^*G12D/*+^ mice were treated with 4-OHT (200 nM) for three days or were left as vehicle treated controls. Microarrays were generated and analyzed with a GSEA and transcription factor signature of the C3 MSigDB collection (version c3.tft.v7.5.1) and the GSEA application (version 4.2.3). Shown is the normalized enrichment score and the q value is color coded. All signatures with a *q* < 0.05 were depicted. Data can be accessed via EMBL-EBI ArrayExpress Accession number: E-MTAB-2592. **c** Upregulation of AP1 family members mRNA upon activation of *Kras*^*G12D*^. Heatmap of AP-1 transcription factor family factors. Shown is the fold induction of the respective JUN and FOS family members upon activation of *Kras*^*G12D*^. Data derived from Microarrays used in (B). **d** Increased AP1 DNA binding over time upon activation of *Kras*^*G12D*^. PDECs from *R26*^*CreERT2*^*;LSL-Kras*^*G12D/*+^ mice were treated with 4-OHT (200 nM) over time as indicated or were left as vehicle-treated controls. Non-radioactive electrophoretic mobility shift assay with a double-stranded oligonucleotide containing an AP1 consensus binding site is shown. **e** FRA1 protein expression is upregulated upon *Kras*^*G12D*^ activation. Western blot of FRA1 expression in PDECs from *R26*^*CreERT2*^*;LSL-Kras*^*G12D/*+^ mice treated with 4-OHT (200 nM) over time or left as vehicle treated controls. β-actin: loading control. *n* = 4. **f** Relative *Fra1* mRNA expression in 4-OHT (200 nM)-treated PDECs was determined by qPCR using *cyclophilin A* mRNA expression as reference (*n* = 4). One-way ANOVA *p* < 0.05

We have recently shown that PDECs isolated from *R26-Cre*^*ERT2*^*;LSL-Kras*^*G12D/*+^ mice allow for the temporally controlled expression of oncogenic Kras^G12D^ driven from the endogenous promoter in the pancreatic context by treatment with 4-hydroxytamoxifen (4-OHT) [25]. In this system, activation of the Cre recombinase induces recombination of the *Kras* locus (Fig. 1a) and activation of canonical KRAS-ERK signaling [25]. To identify genes induced by Kras^G12D^, we analyzed transcriptome profiles of PDECs after three days of treatment with 4-OHT and recently identified an EGFR signaling loop needed for proliferation [3, 25]. In a transcription factor-centered gene set enrichment analysis (GSEA, C3 TFT), we furthermore detected signatures of the AP1 transcription factor family induced by the oncogenic KRAS signal (Fig. 1b). The AP1 transcription factor core family includes the JUN (JUN, JUNB, and JUND) and the FOS (FOS, FOSB, FRA1, and FRA2) family [55–57]. Analyzing signatures from the pathway interaction database (PID), we observed the ERBB network and MYC activity linked to the KRAS signal, corroborating our findings from 2016 [25]. Since we observed the FRA pathway (Supplementary Fig. S1a) and the *Fra1* mRNA is induced by Kras^G12D^ (Fig. 1c), we focused on this AP1 family member. In non-radioactive electrophoretic mobility shift assays using an AP1 consensus-binding site containing double-stranded oligonucleotide, we detected that Kras^G12D^ expression increased AP1 binding over time (Fig. 1d). In Western blots and quantitative PCR analysis, expression of Kras^G12D^ in PDECs induced FRA1 protein and mRNA expression (Fig. 1e, f) over time, corroborating the transcriptome profile data.

### High expression of FRA1 in murine PDACs

To investigate expression of FRA1 in established murine PDACs, we used IHC and Western blots. We observed a gradual upregulation of FRA1 protein expression starting from normal murine PDECs, over pre-neoplastic PDECs isolated from 3 to 6 months old *Ptf1a*^*Cre/*+^*;LSL-Kras*^*G12D/*+^ mice (*KC* afterwards) to murine PDAC cell lines (Supplementary Fig. S1b). Please note that a pure *KC* model is used in all experiments of the manuscript. To demonstrate the pre-neoplastic character of murine PDECs, we measured *p19*^*Arf*^ mRNA expression, encoded by the *Cdkn2a* locus, which is deleted in the majority of murine PDACs [6]. Whereas *p19*^*ARF*^ mRNA expression is induced in PDECs isolated from pre-malignant stages from *KC* mice, no mRNA expression was detected in murine PDAC cancer cell lines (Supplementary Fig. S1c), demonstrating the important tumor suppressive function of *Cdkn2a* for murine PDAC development and showing that the *Cdkn2a* barrier is not conquered in PDECs isolated at pre-malignant stages. Concordantly with the ex vivo models, gradual upregulation of FRA1 protein and mRNA was also observed in vivo during carcinogenesis of *KC* mice (Supplementary Fig. S1d and S1e). In immunohistochemistry (IHC), we detected FRA1 expression in the nucleus of cells of ADM lesions, in the stromal cells surrounding the pre-neoplastic lesions (Supplementary Fig. S1f) and in murine PDAC cells (Supplementary Fig. S1g). These data demonstrate that Kras^G12D^ induces FRA1 expression during the carcinogenesis in the murine pancreas. Hereby, FRA1 is expressed in Kras^G12D^-induced epithelial and PDAC cells as well as cells of the microenvironment.

### Development of FRA1-deficient PDAC in vivo

To decipher the role of FRA1 in the epithelial compartment in vivo, we used mice with floxed *Fra1* alleles (Supplementary Fig. S2a) [30]. In these mice, the exons coding for the dimerization and the DNA-binding domains of *Fra1* (Exon 3 and 4) are flanked by *loxP* sites. Upon Cre-mediated inactivation of *Fra1*, a GFP reporter gene is spliced to the N-terminus of *Fra1* [30]. In the *Ptf1a*^*Cre*^ line, *Fra1* was specifically deleted in the pancreas (Supplementary Fig. S2b) resulting in expression of the GFP-fusion protein in the whole pancreas (Supplementary Fig. S2c). No obvious phenotype was observed in *Ptf1a*^*Cre/*+^*;Fra1*^*lox/lox*^ mice (Supplementary Fig. S2d). When crossed with the *KC* mice, *Ptf1a*^*Cre/*+^*;LSL-Kras*^*G12D/*+^*;Fra1*^*lox/lox*^ mice (*KCF* afterwards) developed ADM and PanIN lesions (Supplementary Fig. S2e and S2f). At 6 months, more low-grade murine PanIN 1A/1B lesions were detected in *KCF* mice (Supplementary Fig. S2f). Like the *KC* mice, *KCF* mice progressed to murine PDACs with a median survival of 489 days (Supplementary Fig. S2g and S2h), which is similar to the survival data for our published *KC* cohort (466 days) [58]. Morphology of *KC* and *KCF* PDACs was not different. Together, these data demonstrate that FRA1 is dispensable for PanIN progression and PDAC formation in a murine model driven solely by Kras^G12D^.

### Human PDACs with high FRA1 enrich pathways associated with basal-like PDACs

**Fig. 2:**
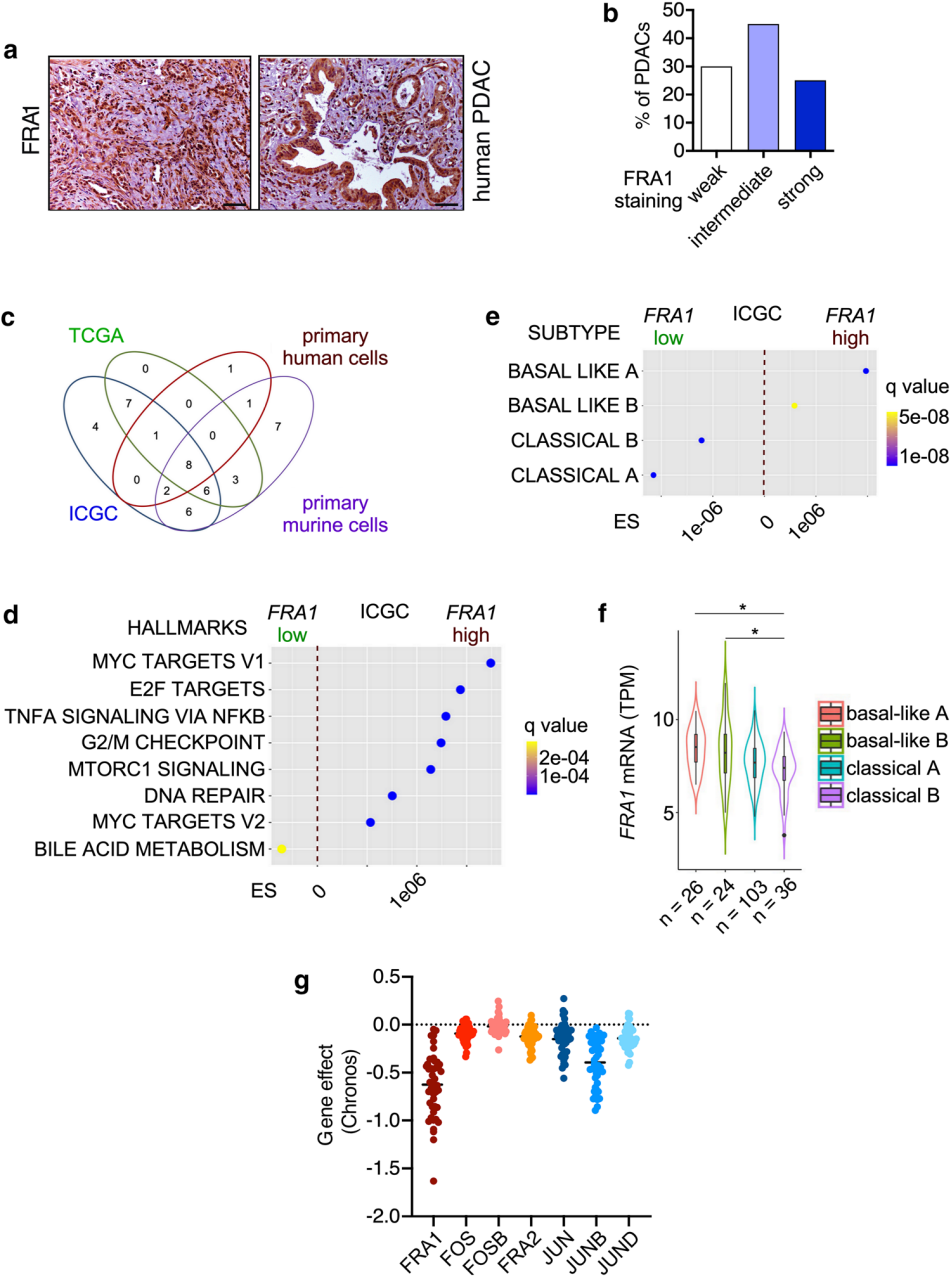
FRA1 is connected to the basal-like subtype of PDAC. **a** Immunohistochemistry of FRA1 in two human PDACs with strong FRA1 expression (scale bar 50 µm). **b** 20 human PDACs were scored for FRA1 expression. Depicted is the fraction of PDACs (%) with weak, moderate, and strong FRA1 expression. **c** Venn diagram showing the overlap of HALLMARK signatures enriched or depleted in PDACs with high *FRA1* mRNA expression (> 75th percentile). mRNA expression datasets of PDAC from the TCGA and ICGC databases as well as primary murine and primary human PDAC cells were analyzed. **d, e **Displayed are the common HALLMARK gene signatures consistently enriched or depleted in PDACs with high *FRA1* mRNA expression corresponding to C and exemplified for the ICGC dataset. **e** Gene signatures corresponding to genes defining classical-A, classical-B, basal-like A, and basal-like B were used for a GSEA. Enrichment scores and q-values for the ICGC dataset are displayed. **f** *FRA1* mRNA expression analysis in PDAC patients divided into classical A, classical B, basal-like A, and basal-like B subtypes. *adjusted *p*-value < 0.05. **g** CRISPR/Cas-drop out screen data were accessed via the DepMap portal. Shown are the gene scores for the core AP1 family and the PDAC context (*n* = 46)

In human PDAC, FRA1 was connected to a worse overall survival [59]. Consistently, we detected a subset of 25% of human PDAC with very high FRA1 expression (Fig. 2a, b). To investigate pathways active in cancers with high FRA1 expression, we used murine (murine PDAC cell lines [6]) and human (primary patient-derived cell lines [47], TCGA PDAC, and ICGC PDAC [45]) mRNA expression datasets. We compared transcriptome profiles of PDACs with high and low *FRA1* mRNA expression with a GSEA using the HALLMARK signatures via the GeneTrail3 platform [48]. In a Venn analysis of all datasets, we observed eight common HALLMARK signatures differentially enriched, with seven HALLMARK signatures enriched and one being depleted in PDACs with high *FRA1* mRNA expression (Fig. 2c, d). These include pro-proliferative E2F, MYC, cell cycle, DNA repair, and inflammatory signatures, which were recently linked to more aggressive cancers [45, 60]. When we used classifier genes indicating classical or more aggressive basal-like cancers as signatures for a GSEA [4], basal-like A and B signatures enrich in PDACs with high *FRA1* mRNA expression (Fig. 2e). These data are consistent with a recently described AP1-driven basal-like PDAC subtype [61] and point to a role of FRA1 in this aggressive subtype. Consistently, in an additional large mRNA expression dataset [4], higher *FRA1* mRNA expression was observed in basal-like A and basal-like B cancers (Fig. 2f). In addition, we accessed human single-cell RNA-seq data from the human PDAC single-cell atlas, which was built from six datasets [50]. We observed high *FRA1* mRNA expression in a subset of ductal cell type 2 cells, which refer to malignant epithelial cells (Supplementary Fig. S3a). Again, we observed a connection between *FRA1* mRNA expression and basal-like cell identity (Supplementary Fig. S3b). To further underscore the relevance of FRA1 in PDAC, we accessed data from a recent CRISPR/Cas-drop out screen [54] for the core AP1 family via the DepMap portal. Indeed, the mean gene effect value was lowest for FRA1 (Fig. 2g), signifying the dependency of a PDAC subtype on FRA1 and thus indicating the need for a more detailed analysis of this particular AP1 family member. Furthermore, such data on the one hand are well in line with the definition of FRA1 as a PDAC-specific “priority therapeutic target” based on another CRISPR/Cas-drop out screen [62], but also point to a specific context, since a strong FRA1 gene effect (< − 1) was evident only in a fraction of PDAC lines.

### Cell autonomous function of FRA1 in PDAC

Since the FRA1 expression data in human and murine PDAC argue for a relevant function of FRA1, we used *Fra1*-deficient murine PDAC cells ex vivo to investigate cell-autonomous roles of FRA1. We were able to isolate three murine PDAC cell lines from *KCF* mice. All three investigated *Fra1*-deficient PDAC cell lines revealed complete recombination of the *Fra1* locus (Supplementary Fig. S4a) and expressed the FRA1-GFP fusion protein (Supplementary Figure S4b). A *Fra1* exon 3 and 4 specific qPCR demonstrated the complete loss of *Fra1* expression in these cells compared to the proficient controls (Supplementary Fig. S4c). *Fra1* exon 1 and 2 specific qPCR showed upregulation of the remaining exons (Supplementary Fig. S4d), suggesting that oncogenic signaling in these PDAC cells induces a compensatory regulatory circuit.

**Fig. 3:**
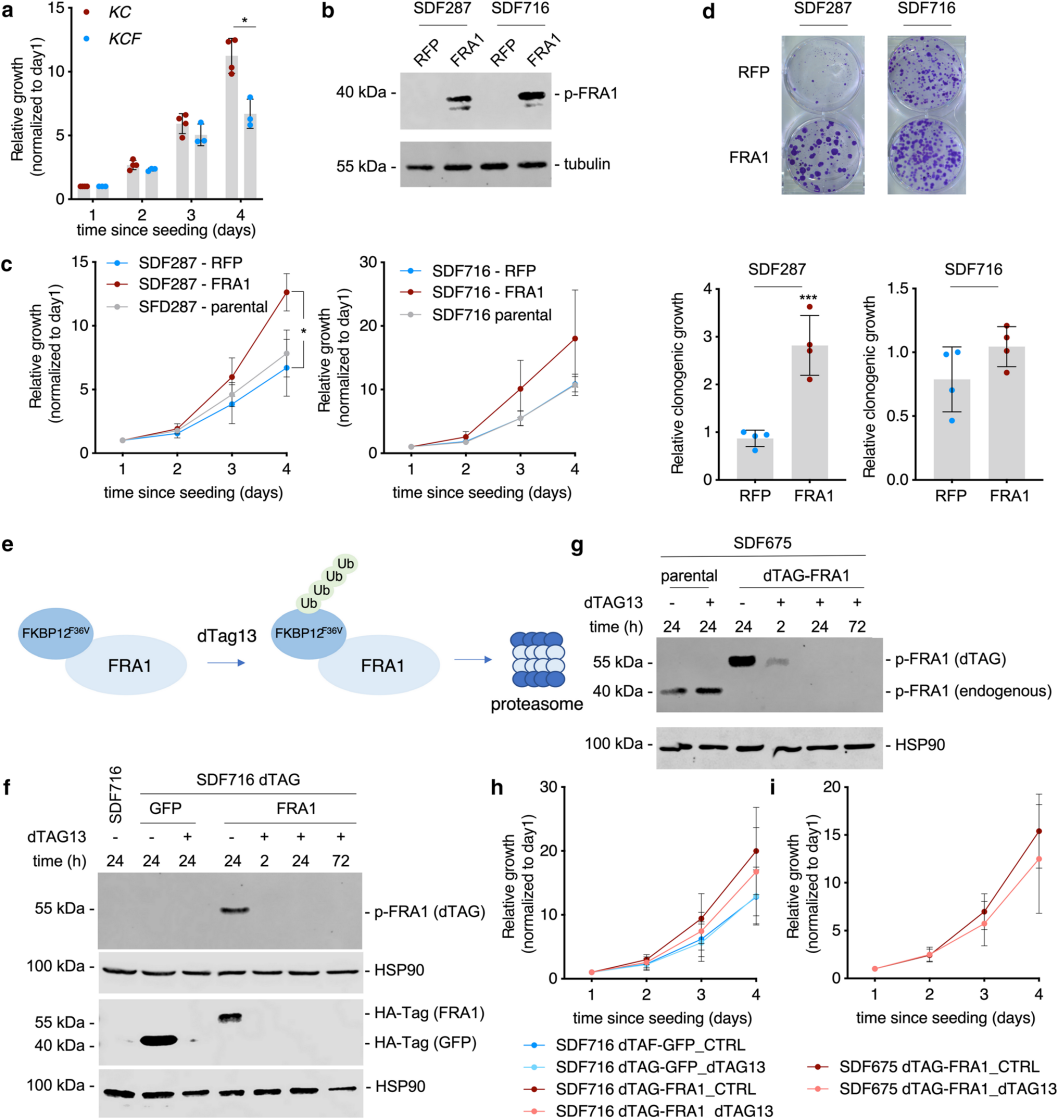
*Fra1*-deficient PDAC cells display impaired growth. **a** Impaired growth in *Fra1*-deficient PDAC cell lines. Relative growth of four *Ptf1a*^*Cre/*+^;*LSL-Kras*^*G12D/*+^ (*KC*) and three *Ptf1a*^*Cre/*+^*;LSL-Kras*^*G12D/*+^*;Fra1*^*lox/lox*^ (*KCF*) PDAC cell lines was determined in MTT assays. 1,000 cells per well of a 96-well plate were seeded out in triplicates. The OD values of each cell line were determined on day 1, 2, 3 and 4 after seeding and are displayed as relative values normalized to day 1. Each dot represents the mean value of one cell line from *n* = 3 biological experiments for all cell lines except for the *KC* cell line PPT-8024 (*n* = 2). The *p*-value of an unpaired Student`s *t*-test at day 4 is indicated. **b** Constitutive reconstitution of FRA1 expression in *KCF* PDAC cells. Western blot with anti-phospho-FRA1 (Ser265) antibody of the KCF cell lines SDF287 and SDF716 transduced either with pLenti-RFP or pLenti-FRA1 vector. Tubulin served as loading control. One representative Western blot out of two independent experiments is shown. **c** Cell growth of two *Ptf1a*^*Cre/*+^*;LSL-Kras*^*G12D/*+^*;Fra1*^*lox/lox*^ (KCF) PDAC cell lines SDF287 (left panel) and SDF716 (right panel) upon reconstitution of FRA1 (pLenti-FRA1) compared to RFP-reporter (pLenti-RFP) transduced or parental cell line was determined in MTT assays. 1,000 cells per well of a 96-well plate were seeded out in triplicates. The OD values of each cell line were determined on day 1, 2, 3 and 4 after seeding and are displayed as relative values normalized to day 1 (mean ± SD from at least three independent experiments). *P-value from ANOVA ≤ 0.05. **d** Clonogenic growth of the two *Ptf1a*^*Cre/*+^*;LSL-Kras*^*G12D/*+^*;Fra1*^*lox/lox*^ (*KCF*) PDAC cell lines SDF287 and SDF716 upon reconstitution of FRA1 (pLenti-FRA1) compared to RFP-reporter (pLenti-RFP) transduced cell lines was determined in clonogenic assays. 2000 cells/well were seeded in 6-well plates in technical triplicates. Upper panel: One representative clonogenic assay out of four independent experiments is shown. Lower panel: Quantification of the clonogenic assays. Crystal violet stainings were solubilized with 1% SDS and OD values measured. Displayed are the mean ± SD from the relative OD values (normalized to pLenti-RFP, arbitrarily set to 1) from four independent experiments (seeded out in technical triplicates). Each dot represents the mean value of the three technical replicates from one experiment. ***P-value of an unpaired *t*-test ≤ 0.001. **e** Schematic description of the dTAG-FRA1 System. Treatment with the dTAG13 degrader allows for selective degradation of mutant FKBP12^F36V^-FRA1 fusion. **f** Selective degradation of dTAG-FRA1 and dTAG-GFP fusion proteins. Western blot with anti-phospho-FRA1 (Ser265) and anti-HA-Tag antibodies of the KCF PDAC cell line SDF716 transduced either with a FKBP12^F36V^-FRA1 (dTAG-FRA1) or a fluorescent control FKBP12^F36V^-GFP (dTAG-GFP) construct. Cells were seeded and treated on the following day with 0.5 µM dTAG13 for the indicated time points to induce selective degradation of the dTAG-GFP or dTAG-Fra1 fusion proteins. The dTAG-constructs contain an HA-Tag allowing for immunodetection with an anti-HA-Tag antibody. The *Fra1*-deficient SDF716 parental wild-type (wt) cell line served as a control. HSP90 was used as a loading control. **g** Selective degradation of dTAG-FRA1 fusion protein. Western blot with anti-phospho-FRA1 (Ser265) antibody of the *Pdx1-Flp;FSF-Kras*^*G12D/*+^*, p53*^*frt/*+^*; Fra1*^*lox/lox*^ cell line SDF675 transduced with an inducible Cre recombinase (pInducer-iCre) and a FKBP12^F36V^-FRA1 (dTAG-FRA1) construct. After treatment for 8 days with 100 ng/mL Doxycycline to induce the Cre-Recombinase and subsequent recombination of the endogenous floxed *Fra1* alleles, cells were seeded and treated on the following day with 0.5 µM dTAG13 for the indicated time points to induce selective degradation of the dTAG-FRA1 fusion protein. The parental wild-type (wt) cell line served as a control for the expression of endogenous FRA1. HSP90 was used as a loading control. **h** Relative growth of the *Ptf1a*^*Cre/*+^*;LSL-Kras*^*G12D/*+^*;Fra1*^*lox/lox*^ (*KCF*) PDAC cell line SDF716 transduced either with dTAG-FRA1 or dTAG-GFP vectors. For each condition, 1000 cells were seeded per well and treated on the following day with 1 µM dTAG13 to induce degradation of the dTAG fusion protein. Relative growth was determined by MTT assays. The absorbance values of each cell line were determined on day 1, 2, 3 and 4 after seeding and are displayed as relative values normalized to day 1 (mean ± SD from three independent experiments). **i** Relative growth of the *Pdx1-Flp;FSF-Kras*^*G12D/*+^*, p53*^*frt/*+^*; Fra1*^*lox/lox*^ PDAC cell line SDF675 transduced with dTAG-FRA1 and pInducer-iCre vectors. Cells were treated for 8 days with doxycycline to induce complete recombination of the endogenous floxed Fra1 allele. For each condition, 1,000 cells were seeded per well in triplicates and treated on the following day with 1 µM dTAG13 to induce degradation of the dTAG-FRA1 fusion protein. Relative growth was determined by MTT assays. The OD values of each cell line were determined on days 1, 2, 3, and 4 after seeding and are displayed as relative values normalized to day 1. Data are presented as mean ± SD from three independent experiments

To further elucidate FRA1 functions, we determined growth. Compared to four *Fra1*-proficient murine PDAC cell lines, growth of the three investigated *Fra1*-deficient lines was significantly decreased 96 h after seeding (Fig. 3a), arguing for a cell-autonomous role of FRA1 in the regulation of growth/proliferation ex vivo. Using the diameter of spheroids as a read-out for growth, impaired growth of *Fra1*-deficient murine PDAC cells was also observed under 3D culture conditions (Supplementary Fig. S4e). To decipher the role of FRA1 in regulation of growth in more detail, we reconstituted FRA1 expression in the *Fra1*-deficient *KCF* cells by transduction with a constitutive FRA1 expression construct (pLenti-FRA1) or their respective control vector expressing an RFP reporter (pLenti-RFP) (Fig. 3b). FRA1-specific antibodies that we have tested all target the N-terminus and recognized both FRA1 and the FRA1-GFP fusion protein. Since both have similar molecular weights, it was impossible to differentiate between *Fra1*-defcient and reconstituted cells by Western blotting. Phosphorylation of FRA1 on Ser265 stabilizes the protein, and a phospho-FRA1-specific antibody was able to differentiate between *Fra1*-deficient and *Fra1*-reconstituted cells (Fig. 3b). To different degrees, cell growth in the two *KCF* cell lines was increased upon reconstitution of FRA1 compared to the parental cell line and the mock-transduced control (Fig. 3c). Clonogenic growth was also significantly higher upon reconstitution of FRA1 at least in the SDF287 cell line upon reconstitution of FRA1 (Fig. 3d), thus indicating a pro-proliferative role of FRA1 in a subset of PDAC.

Currently, there are no FRA1-specific inhibitors available. To mimic the effect of an acute small-molecule mediated depletion of FRA1, we made use of the dTAG system, which allows for target-specific degradation of mutant FKBP12^F36V^ fused to the protein of interest by the small molecule degrader dTAG13 (Fig. 3e) [35]. Firstly, we transduced the *Fra1*-deficient *KCF* cell line SDF716 with a FKBP12^F36V^-FRA1 (dTAG-FRA1 afterwards) expression construct or an FKBP12^F36V^-GFP (dTAG-GFP afterward) control vector. Treatment with the dTAG13 degrader led to rapid degradation of the dTAG-FRA1 protein chimera within 2 h that lasted for at least 72 h (Fig. 3f). Secondly, we used the dual recombinase system [28] to generate a murine PDAC cell line with two floxed *Fra1* alleles. We transduced SDF675 cells with a doxycycline-inducible iCre recombinase (pInducer-iCre) and the dTAG-FRA1 construct. Treatment with doxycycline for 8 days deleted both *Fra1* alleles (Fig. 3g). Also in this cellular model, rapid degradation of the dTAG-FRA1 protein chimera was observed, occurring within 2 h (Fig. 3g). Growth of the *KCF* cell line SDF716 transduced with dTAG-FRA1 was increased compared to the dTAG-GFP controls (Fig. 3h), again supporting the pro-proliferative function of FRA1 in these models. Whereas dTAG13 did not affect on the dTAG-GFP cells, growth was slightly diminished in the dTAG-FRA1 cells upon dTAG13 treatment (Fig. 3h). Also in the SDF675 dTAG-FRA1 cell line, growth was only slightly lower upon degradation of FRA1 (Fig. 3i). Together, these data argue that—even though FRA1 has pro-proliferative, growth-promoting functions—acute loss of FRA1 can be partially compensated, giving rise to only slightly decreased cell growth in the tested cell lines.

### FRA1 and drug responsiveness of PDAC cells

**Fig. 4:**
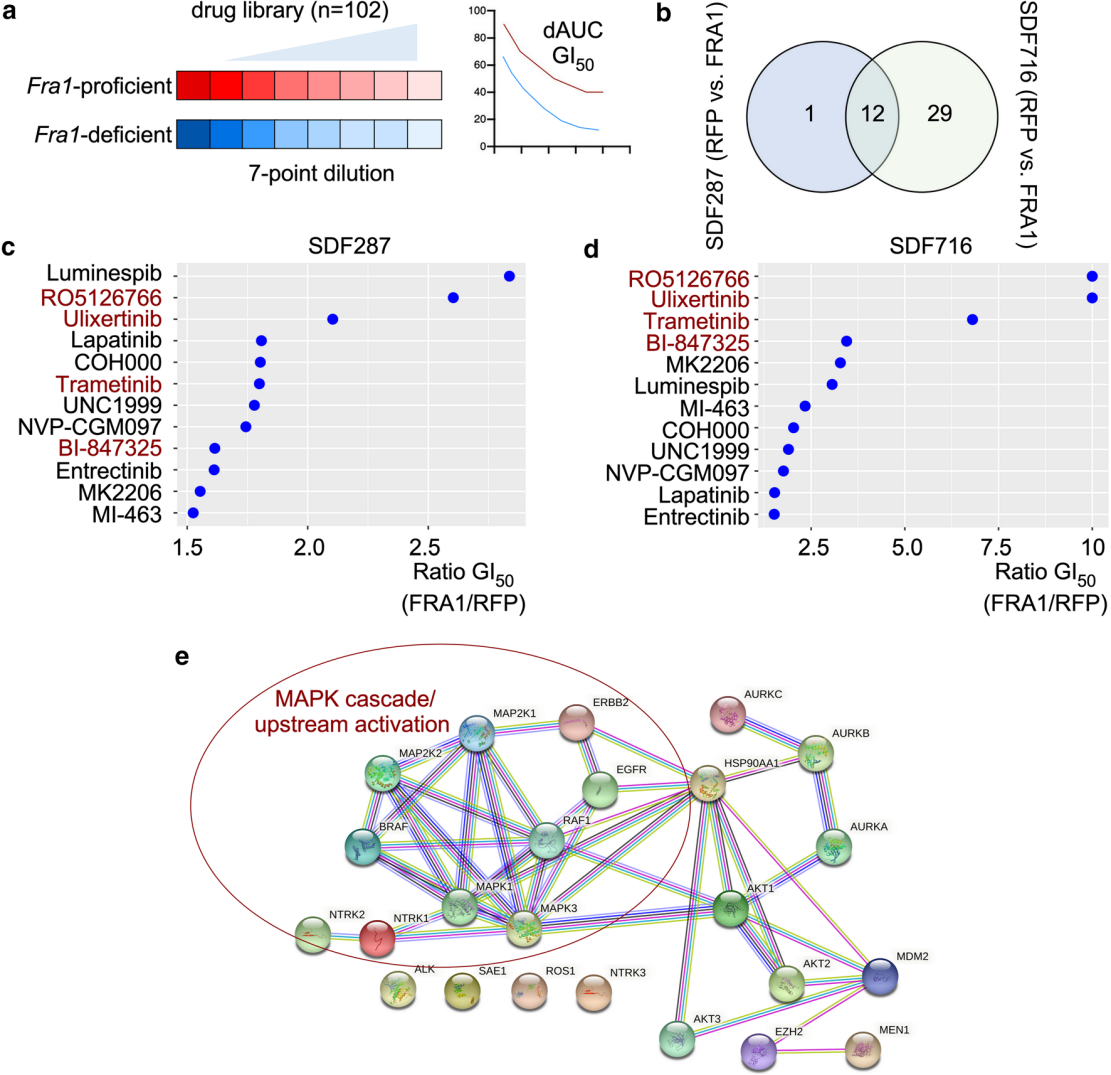
Unbiased drug screen identifies MAPK inhibitors as druggable vulnerabilities of *Fra1*-deficient PDAC cells. **a** Schematic description of the drug screen setup*.* Isogenic *Fra1*-proficient and *Fra1*-deficient cell lines were screened with a drug library consisting of 102 compounds in 7-point serial dilutions. Cell viability after 72 h of treatment was assessed by CellTiter-Glo and GI_50_ and AUC values were calculated. **b** Venn analysis identifies drugs to which both *Fra1*-deficient cell lines were more sensitive to. A GI_50_ ratio (FRA1/RFP) > 1.5 was used as the threshold for differential sensitivity. **c, d** Ratio of GI_50_ (Fra1/RFP) values from the drug screen for the 12 common hits identified in (**B**). Hits are depicted in (**C**) for SDF287 and in (**D**) SDF716 cells. Four out of the twelve drugs are RAF-MEK-ERK cascade inhibitors and are marked in red. Ratios were arbitrarily restricted to a max. of 10. **e** STRING pathway analysis was performed using the targets of the drug screening experiment and the STRING web platform using multi protein modus, full STRING network, and highest confidence interaction score (0.9) as setting. Marked is the canonical KRAS-RAF-MEK-ERK nodus

Since FRA1 has been shown to modulate the drug response of KRAS-driven cancer cells [59], we performed an unbiased drug screening experiment in isogenic models. We used *KCF* cells transduced with RFP or FRA1 (Fig. 3b). We used a drug library consisting of 102 compounds, which target various cancer-relevant pathways (Fig. 4a). Cell viability was determined 72 h after drug administration by measuring ATP as a surrogate for the drug response. AUC and GI_50_ values were calculated based on the GRmetrics methodology [38] (Supplementary Table S1). Calculation of the delta-AUC (AUC (Fra1)—AUC (RFP)) showed that FRA1 confers mainly therapy resistance (Supplementary Fig. S5a). To identify common hits between the two cell lines, we performed a Venn diagram analysis of all drugs with at least 1.5 fold lower GI_50_ value in *Fra1*-deficient cells. Thereby, we identified 12 drugs to which both of the *Fra1*-deficient cell lines were more sensitive than the *Fra1*-reconstituted cells (Fig. 4b–d). A String pathway analysis showed that the hits were centered around the canonical KRAS signaling pathway (Fig. 4e). Indeed, four out of the twelve hits (33%) were targeting the MAPK pathway. These inhibitors include the dual MEK/Aurora kinase inhibitor BI-847325, the dual RAF/MEK inhibitor RO5126766, the MEK1/2 inhibitor trametinib, and the ERK1/ERK2 inhibitor ulixertinib, arguing that FRA1 confers resistance to inhibitors of the entire RAF-MEK-ERK cascade. Of note, no overlap in the two screened cell lines exists for drugs with higher activity in FRA1 re-expressing cells. SDF716 cells were more sensitive to AZD5153 (BET inhibitor), TAK-243 (UAE inhibitor), and BI-D1870 (RSK inhibitor) upon FRA1 re-expression, whereas SDF287 cells were more sensitive to BAY-876 (GLUT1 inhibitor).

In sum, the drug screening experiment points to a role of FRA1 in mediating resistance to inhibitors of the canonical KRAS signaling.

### FRA1 modulates MAPK and AKT signaling

**Fig. 5:**
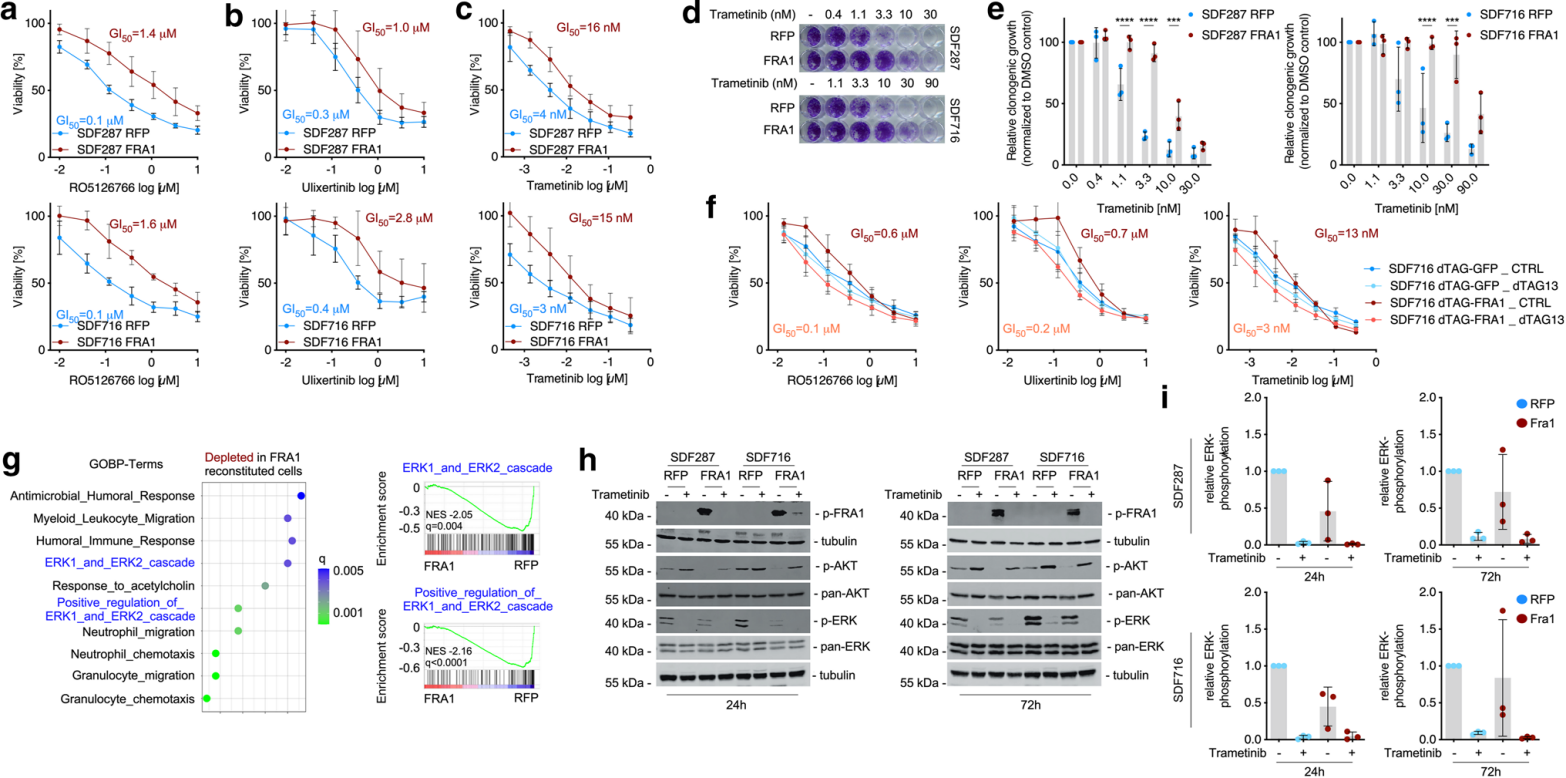
Loss of *Fra1* triggers increased MAPK signaling and sensitivity to MAPK inhibitors. (**a**–**c**) *Fra1*-deficient cells are sensitive to MAPK inhibitors. *Fra1*-deficient and -proficient cells were treated in technical triplicates with 7-point serial dilutions of the dual RAF/MEK inhibitor RO5126766 (**A**), the ERK inhibitor ulixertinib (**B**) or the MEK inhibitor trametinib (**C**). Upper panel: Cell line SDF287, transduced with RFP or FRA1 expression vectors. Lower Panel: Cell line SDF716 transduced with RFP or FRA1 expression vectors. Viability was determined by MTT assay after 72 h of drug treatment and is displayed as relative viability normalized to DMSO treated controls. Values represent the mean of three independent experiments ± SD. GI_50_ values were calculated by non-linear regression and are indicated in the respective figures. **d** Clonogenic growth of *Fra1*-deficient (RFP) and *Fra1*-proficient (FRA1) cell lines upon treatment with the MEK inhibitor trametinib in the two cell lines SDF287 (upper panel) and SDF716 (lower panel). Cells (1000/well) were seeded out in 24-well plates, treated on the following day with the indicated doses of trametinib in technical duplicates and stained with crystal violet after 7 days of treatment. One representative image from three independent experiments is shown. **e** Quantification of the clonogenic assays shown in (**D**). Crystal violet stainings were solubilized in 1% SDS and absorbance was measured in a microplate reader. Absorbance values were normalized to DMSO treated controls and are displayed as relative clonogenic growth. Each dot represents the mean value (from the technical duplicates) of one independent experiments. Data are presented as mean ± SD from three independent experiments. Left panel: SDF287. Right panel: SDF716. P-value of a 2way ANOVA with multiple comparisons. ***P ≤ 0.001. ****P ≤ 0.0001. **f** Perturbation of FRA1 sensitizes to MAPK inhibition. The *Fra1*-deficient KCF cell line SDF716 was reconstituted with the dTAG-FRA1 construct (or dTAG-GFP as control). SDF716 dTAG-FRA1 and SDF716 dTAG-GFP cells were seeded out in 96-well plates (1,000/well) in growth medium containing either 1 µM dTAG13 to induce degradation of the dTAG-fusion protein or DMSO as control. On the following day, the cells were treated in technical triplicates with 7-point serial dilutions of the dual RAF/MEK inhibitor RO5126766 (left panel), the ERK inhibitor ulixertinib (middle), or the MEK inhibitor trametinib (right panel). Viability was determined by MTT assay after 72 h of drug treatment and is displayed as relative viability normalized to vehicle-treated controls. Values represent the mean of three independent experiments ± SD. GI_50_ values were calculated by nonlinear regression and are indicated in the respective figures. **g** ERK1/2 gene signatures are enriched in *Fra1*-deficient cells. RNA-seq with subsequent geneset enrichment analysis was performed in *Fra1*-deficient (SDF287 RFP and SDF716 RFP) and *Fra1*-reconstituted cells (SDF287 FRA1 and SDF716 FRA1). Left panel: Shown are the top ten gene signatures from the Gene Ontology Biological Process (GOBP) database that are depleted in the two FRA1-reconstituted cell lines compared with *Fra1*-deficient controls. Normalized enrichment scores and q-values are shown. Right panel: Enrichment plot for the ERK1/2 cascade-related gene signatures “ERK1 and ERK2 cascade” (upper) and “positive regulation of ERK1 and ERK2 cascade” (lower). **h** Western blots for phospho-ERK and phospho-AKT upon treatment with trametinib after 24 h (left panel) and 72 h (right panel) in *Fra1*-deficient and -proficient cells. **i** Quantification of ERK phosphorylation in *Fra1*-proficient and -deficient cell lines. Depicted is the ratio of phospho-ERK to total ERK from the Western blots in H). Left panel: SDF287 cell line (24 and 72 h trametinib treatment); right panel: SDF716 cell line (24 and 72 h treatment). Each dot represents one independent experiment

Due to a connection of FRA1 to resistance to canonical KRAS signaling inhibitors, we focused on this pathway for further analysis. Consistent with the PDEC data and direct KRAS inhibitors [63, 64], *Fra1* mRNA expression is reduced upon the treatment of murine PDAC cells with MEK inhibitors (Supplementary Fig. S5b). To validate these findings, we performed viability assays with the inhibitors RO5126766, ulixertinib, and trametinib (Fig. 5a–c). Confirming the drug screen, *Fra1*-deficient cells were more sensitive to these inhibitors compared with FRA1-reconstituted ones. GI_50_ values were around three- to sevenfold lower in the *Fra1*-deficient cells for trametinib and ulixertinib, and more than tenfold lower for RO5126766. Additionally, *Fra1*-deficient cells were also more sensitive to trametinib in long-term clonogenic growth assays (Fig. 5d, e).

To determine whether acute depletion of FRA1 affects the drug response to MAPK inhibitors, we treated dTAG-FRA1 transduced cells with the dTAG13 degrader. Analogously to the reconstitution of FRA1 in these cells, expression of the dTAG-FRA1 in SDF716 PDAC cells rendered the cells more resistant to the three MAPK inhibitors compared with dTAG-GFP controls (Fig. 5f). Degradation of the dTAG-FRA1 fusion protein by dTAG13 sensitized the cells to MAPK inhibition, with three- to sixfold changes in GI_50_ values. In contrast, no such effect was observed upon dTAG13 treatment of the GFP-dTAG control cells.

Also in the SDF675 dTAG-FRA1 cell line, in long-term clonogenic assays, growth upon trametinib treatment was significantly lower in the dTAG13-treated, FRA1-degraded cells (Supplementary Fig. S5c and S5d). Together, these data indicate that even acute depletion of FRA1 can also sensitize PDAC cells to MAPK inhibition.

To decipher the molecular functions that determine the role of FRA1 in mediating resistance to MAPK inhibition, we performed RNA-seq of *Fra1*-deficient and FRA1-reconstituted cells. We used gene set enrichment analysis and GO-terms to define pathways regulated by FRA1. We found that FRA1 represses signatures linked to immune regulation (Fig. 5g). Furthermore, signatures indicative of a function of FRA1 in the regulation of the MAPK signaling cascade were detected and found to be depleted in FRA1-reconstituted PDAC cells (Fig. 5g). Since the regulation of the ERK cascade might explain the altered sensitivity toward ERK, RAF, and MEK inhibitors, we investigated the signaling pathway by using ERK phosphorylation as a surrogate for activity. We treated *Fra1*-proficient and *Fra1*-deficient cells with the MEK1/2 inhibitor trametinib for 24 and 72 h and determined MAPK and PI3K pathway activity (Fig. 5h). As expected, treatment with trametinib strongly decreased phosphorylation of ERK after 24 and 72 h of treatment (Fig. 5h, i). The downregulation was observed both in the *Fra1*-deficient and in the *Fra1*-proficient cells. Whereas phosphorylation of ERK was decreased upon MEK inhibition, phosphorylation of AKT was increased by trametinib treatment (Fig. 5h, i). However, also here, no difference in upregulation of phospho-AKT was observed. Importantly, a marked difference was detected in the basal ERK phosphorylation levels between the untreated controls. Compared with the FRA1-reconstituted cells, *Fra1*-deficient cells had distinctly higher levels of phospho-ERK (Fig. 5h, i), which is consistent with the GSEA data (Fig. 5g). *Fra1*-deficient cells also displayed higher levels of AKT phosphorylation (Fig. 5h), indicating that perhaps both the MAPK and the PI3K pathways are needed to compensate for the loss of FRA1. In the dTAG-FRA1 model, the increased phosphorylation of ERK—but not AKT—upon loss of FRA1 was already observed after 24 h of dTAG13 treatment, arguing for a rapid rewiring of oncogenic signaling pathways (Supplementary Fig. S5e). Since we observed a rather variable contribution of FRA1 to growth and sensitivity to MAPK inhibitors, we established an additional genetic model, allowing the doxycycline-dependent induction of FRA1 in *KCF* cell lines. FRA1 was induced in three lines by low-dose doxycycline treatment (Supplementary Fig. S6a). However, the induction of FRA1 in these lines over four days did not change their growth (Supplementary Fig. S6b). We additionally investigated the responsiveness of these lines to inhibitors of MAPK signaling in the presence and absence of doxycycline. Although the GI_50_ was increased in all models and for all inhibitors tested in the FRA1 “on” setting, the effects were again highly context dependent and ranged from a small twofold to a rather distinct 11–26-fold increase in the GI_50_ values (Supplementary Fig. S6c). In addition, we investigated RNA-seq data of doxycycline-treated cells 24 h after inducing FRA1 expression. Overall, the changes of mRNAs were again context dependent and rather mild. Although the line with the strongest depletion of the GO-term signatures linked to ERK signaling upon FRA1-induction revealed the greatest change in MAPK inhibitor GI_50_ values, all *q*-values were > 0.05 (Supplementary Fig. S6d). Furthermore, the influence of FRA1-induction on ERK phosphorylation was not clearly observed in the doxycycline-inducible model (Supplementary Fig. S6e). Collectively, these data show highly context-dependent functions of FRA1 in the regulation of PDAC growth and a contribution to the control of sensitivity toward inhibitors of the MAPK signaling pathway.

## Discussion

To find transcription factors linked to oncogenic KRAS signal transduction in pancreatic cancer, we used primary pancreatic ductal epithelial cells and identified the AP1 transcription factor programs to be activated by oncogenic KRAS. The AP1 complex recognizes specific sequences in promoters and enhancers of target genes and controls critical aspects of tumorigenesis, like cell proliferation or survival/apoptosis [56].

Here, we focused on the AP1-family member FRA1 [55, 57] and demonstrate that FRA1 is highly expressed in a subset of PDACs, but dispensable for murine Kras^G12D^-induced PanIN formation and PDAC development. The important role and functions of FRA1 in pancreatic cancer carcinogenesis as well as in tumor progression have been highlighted recently. In FRA1 gain-of-function models, the impact of the transcription factor to promote acinar-to-ductal metaplasia development, an early step in pancreatic carcinogenesis, was demonstrated [59]. Consistent with Vallejo et al. [59], we found increased expression of FRA1 in pre-neoplastic lesions as well as a PDACs tissue and cells compared with the normal pancreas. In addition, we observed a connection of FRA1 to basal-like cancers, which is consistent with the worse prognosis linked to both FRA1-active and basal-like PDACs [59, 60, 65]. Basal-like A cancers reveal a higher metastatic probability [4]. Consistently, a role of FRA1 in PDAC metastasis models was shown [65], which underscores the relevance of FRA1 in PDAC.

In addition to the epithelial compartment, we detected FRA1 expression also in stromal cells of the tumor microenvironment. Up to 90% of PDAC is stroma, composed of an extracellular matrix hosting stellate cells, tumor-associated fibroblasts, immune cells, nerves, and vessels. This argues for important functions of FRA1 in cells like cancer-associated fibroblasts, which should be deciphered in future work.

Recent work showed that FRA1 contributes early to the KRAS-driven carcinogenesis by regulating and maintaining chromatin accessibility at genetic loci needed for carcinogenesis [66, 67]. In KRAS-driven lung cancer or cholangiocarcinoma models, the knock-out of *Fra1* significantly delays tumor development [59, 68, 69]. In a murine PDAC model, dependent on a gene transfer by adeno-associated virus (AAV) serotype 8, in which AAV8 was used to deliver Cre together with multiplexed sgRNAs, targeting *Cdkn2a*, *Trp53*, *Smad4*, and *Fra1*, to the pancreas *of LSL-Kras*^*G12D*^*;LSL-Cas9-eGFP* mice, the impact of FRA1 to early steps in the carcinogenesis was demonstrated [67]. However, in our investigated Kras^G12D^-driven PDAC in vivo model, FRA1 is dispensable for PanIN progression and PDAC formation. We observed no difference in ADM formation between *KC* and *KCF* mice and *Fra1*-deficient mice developed PDAC with a comparable survival rate to *KC* mice. Such data demonstrate the relevance of the oncogenic context for the KRAS-driven carcinogenesis [70]. Whether redundant functions in the AP1 family, cooperating tissues specific transcription factors, alternative genetic trajectories, re-wired oncogenic signaling, or non-cell-autonomous processes explain the compensation of FRA1 for tumor formation is unclear and demands additional investigations.

Nevertheless, *Fra1*-deficient PDAC cells established from KCF mice displayed variable growth defects in vitro. Furthermore, several genetic gain- and loss-of-function models showed that—to different degrees and dependent on the mode of FRA1 re-expression—FRA1 can augment the growth of PDAC cells. These observations are consistent with a function of FRA1 in the regulation of the cell cycle and effects toward the G1-phase or the G2/M-phases of the cycle have been described [59, 69, 71]. Furthermore, such mechanistic data are in line with the association of FRA1 with pro-proliferative pathways in PDAC mRNA expression datasets. However, the data also underscore a highly context-dependent function of FRA1, a note which is also supported by the CRISPR/Cas9-drop out screens. In such screens, strong FRA1 gene effects were observed only in a fraction of PDAC cell lines.

In cancer cells driven by oncogenic KRAS, genes relevant for G2/M-phases of the cell cycle, like Aurora A kinase, were found to be linked to FRA1 [59] and expose a therapeutic vulnerability. Prompted by this observation, we performed an unbiased drug screen to define FRA1-associated vulnerabilities. Hereby, we observed that *Fra1*-deficient cells are more sensitive to inhibitors of the canonical KRAS signaling pathway, including RAF-, MEK-, and ERK-inhibitors. We detected compensatory rewiring of oncogenic signaling pathways that might at least partially explain the drug screening hits. In particular, we observed a transcriptional and post-translational upregulation of the MAPK pathway in constitutive FRA1-knockout cells as well as upon small-molecule-induced degradation of FRA1. Re-expression of FRA1 renders KRAS-driven PDACs cells more independent of canonical signaling, underpinning the note that FRA1 may maintain major parts of the transcriptional output of oncogenic KRAS. Such insights are consistent with data elaborated in a switchable *Kras*^*G12D*^ model, where FRA1 transfers tumor-forming capabilities in the *KRAS* “off” setting, demonstrating that the transcription factor can substitute signaling by the oncogene [67]. Furthermore, a recent genome-scale CRISPR-Cas9 screen in KRAS-mutant pancreatic or lung cancer cell lines in the presence or absence of the MEK inhibitor trametinib unveiled *FRA1* knock-out as a sensitizer to MEK inhibition in 2 out of 5 pancreatic cancer cell lines, but none of the 5 lung cancer cells [72]. An in vivo CRISPR-Cas9 drop-out screen in one human PDAC patient-derived xenotransplant demonstrated no prominent role of a *FRA1* knock-out as a trametinib sensitizer [73]. In addition to canonical RAF-MEK-ERK signaling inhibitors, direct KRAS inhibitors are under development and KRAS^G12C^ inhibitors are approved for the treatment of lung cancers [7]. Unbiased genetic screening experiments demonstrated that inhibition of KRAS^G12C^ by ARS-1620 renders the human PDAC cell line MiaPaCa-2 dependent on FRA1 [63]. Consistent with the genetic data, we describe here variable effects of FRA1 on the control of MAPK inhibitor sensitivity, which ranged from a 2- to a 26-fold change in GI_50_ values. Therefore, also with respect to the modulation of drug sensitivity, the function of FRA1 is context dependent and it will demand additional efforts to decipher the relevant molecular determinants.

It is important to note that the described molecular circuits were not observed in all models. Although the modulation of the sensitivity toward MAPK pathway inhibitors was consistently observed also in the doxycycline-inducible models, the connection to ERK signatures and phosphorylation was not. Whether a longer time period of constant FRA1 expression is needed to explain the discrepant effects in the investigated models needs further investigation.

Our findings underscore the complex multi-layered buffering of oncogenic signaling in PDAC, where the loss of one signaling node is quickly compensated by the upregulation of another node. These rapid adaptations make mono-targeted therapies challenging and point to the need to decipher the molecular mechanisms behind these relevant therapeutic processes, including a permissive context, in more detail to develop suitable combination therapies.

Taken together, our data indicate a complex and context-dependent role of FRA1 in PDAC. Even though FRA1 is expressed in pre-neoplastic lesions and PDACs compared to the normal pancreas, it seems to be dispensable for PDAC oncogenesis in a murine Kras^G12D^-driven PDAC model. Although this redundancy observed in the in vivo model might argue against the development of a specific FRA1 inhibitor, pharmacological FRA1 inhibition, which we mimic by a chimeric dTAG-FRA1, can render PDAC cells sensitive to RAF-, MEK-, ERK-inhibition, offering opportunities for the development of rational and mechanism-based combination therapies.

### Supplementary Information

Below is the link to the electronic supplementary material.

Supplementary file1 (PDF 11467 KB)

## Data Availability

mRNA expression datasets of *Fra1*-deficient and reconstituted murine PDAC cells can be freely accessed via ENA: PRJEB52257. RNA expression of trametinib-treated murine PDAC cells can be accessed via: E-MTAB-11187.
