# Supplementary material for: AP1/Fra1 confers resistance to MAPK cascade inhibition in pancreatic cancer
Source: Cell Mol Life Sci. 2022 Dec 19;80(1):12. doi: 10.1007/s00018-022-04638-y (PMC9763154; doi:10.1007/s00018-022-04638-y)
Supplement: Supplementary file 1 — Supplementary file1 (PDF 11467 KB) [file 18_2022_4638_MOESM1_ESM.pdf]

Supplementary Figures 1 – 6

AP1/Fra1 confers resistance to MAPK cascade inhibition in pancreatic cancer

Schneeweis et al.

*Correspondence: guenter.schneider@med.uni-goettingen.de

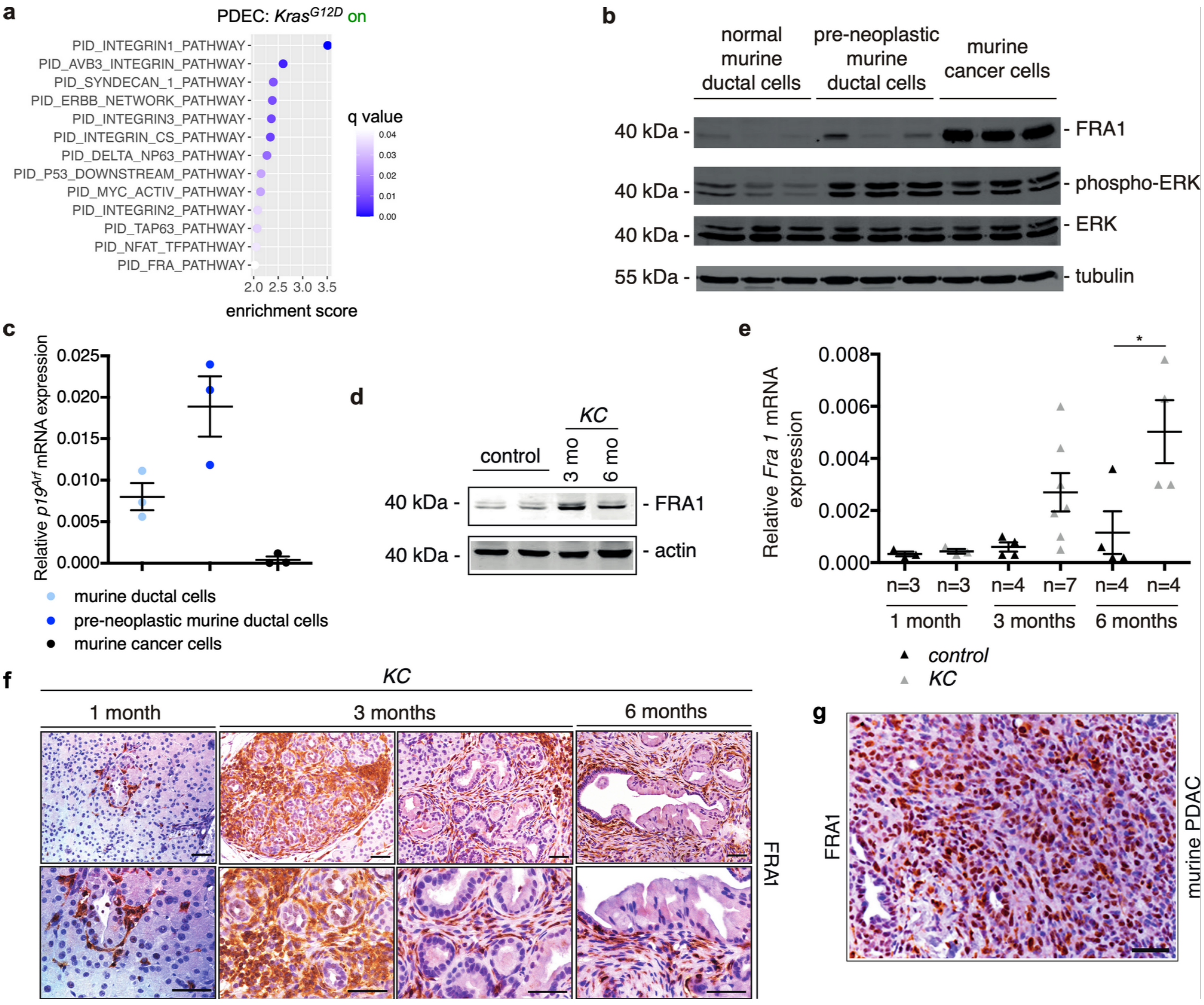

Figure. S1. FRA1 expression is upregulated in PanINs and PDACs.

a PID Gene Sets upregulated upon activation of oncogenic *Kras*^{G12D}. PDECs from *R26*^{CreERT2}*;LSL-Kras*$^{G12D/+}$ mice were treated with 4-OHT (200 nM) for three days or were left as vehicle treated controls. Microarrays were generated and analyzed with a GSEA using the GSEA application version 4.2.3 and the pathway interaction database (PID) gene

sets. Shown is the normalized enrichment score and the q value is color-coded. All signatures with a q<0.05 are depicted. Data can be accessed via EMBL-EBI ArrayExpress Accession number: E-MTAB-2592.

b FRA1 protein expression is upregulated in murine PDAC cells. Western blot of phospho-ERK, pan-ERK and FRA1 expression (α-tubulin: loading control) in PDECs, pre-neoplastic murine ductal cells and PDAC cells. PDECs were isolated from control mice (normal murine ductal cells; n=3) and from 3-6 month old *Ptf1a*$^{Cre/+}$;*LSL-Kras*$^{G12D/+}$ (*KC*) mice (pre-neoplastic murine ductal cells; n=3). Furthermore murine PDAC cell lines from *Ptf1a*$^{Cre/+}$;*LSL-Kras*$^{G12D/+}$ (*KC*) mice were used (n=3).

c Relative p19^{Arf} mRNA expression in murine ductal cells, pre-malignant cells and cancer cells was determined by qPCR using cyclophilin A mRNA expression as reference. Each dot represents expression in one cell line.

d Increased FRA1 protein expression in the pancreas of KC mice. Western blot of FRA1 expression in 3 and 6 month old *Ptf1a*$^{Cre/+}$;*LSL-Kras*$^{G12D/+}$ (*KC*) mice compared to two wild type mice (β-actin: loading control).

e Upregulated *Fra1* mRNA expression in the pancreas of 3 and 6 months old KC mice. Relative *Fra1* mRNA expression in the pancreas of age matched wild-type and *Ptf1a*$^{Cre/+}$;*LSL-Kras*$^{G12D/+}$ (*KC*) mice was determined by qPCR using *cyclophilin A* mRNA expression as reference.

f FRA1 immunohistochemistry in the pancreas from 1, 3 and 6 months old *Ptf1a*$^{Cre/+}$;*LSL-Kras*$^{G12D/+}$ (*KC*) mice (scale bar 50 µm).

g FRA1 immunohistochemistry of a PDAC from a *Ptf1a*$^{Cre/+}$;*LSL-Kras*$^{G12D/+}$ (*KC*) mouse (scale bar 50 µm).

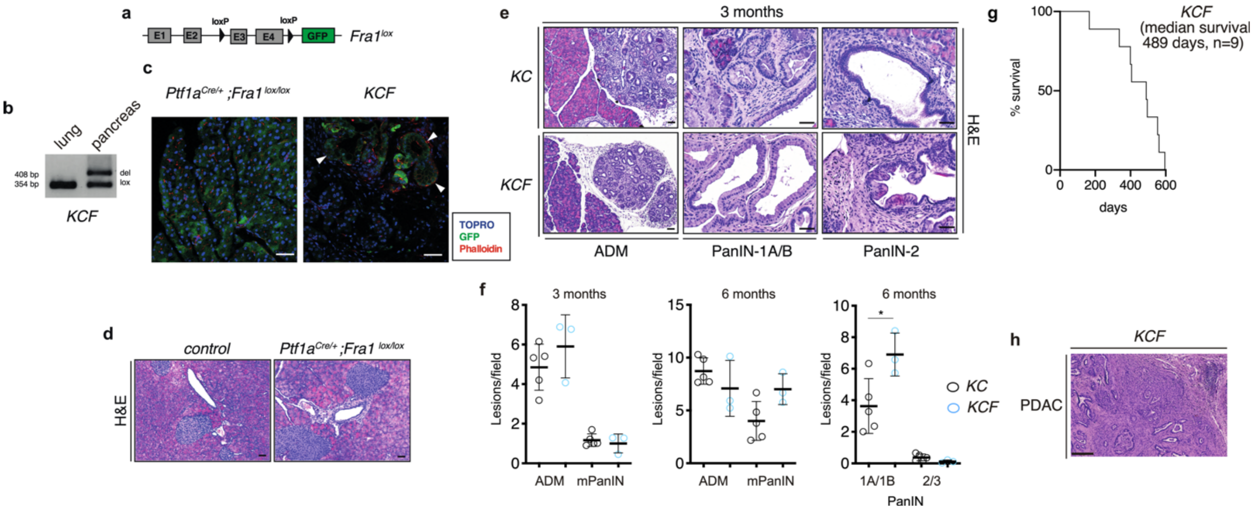

Figure. S2. FRA1 is dispensable for PDAC formation.

a Scheme of the conditional *Fra1^{lox}* allele.

b Pancreas-specific targeting of the *Fra1* allele in KCF mice. *Fra1* genotyping PCR with DNA from lung and pancreas of *Ptf1a$^{Cre/+}$;LSL-Kras$^{G12D/+}$;Fra1$^{lox/lox}$* (*KCF*) mice.

c Fluorescence microscopy of GFP expression in *Ptf1a$^{Cre/+}$;Fra1$^{lox/lox}$* (left) and *Ptf1a$^{Cre/+}$;LSL-Kras$^{G12D/+}$;Fra1$^{lox/lox}$* mice (right). Nuclear staining with TO-PRO-3 (blue) and actin filament staining with phalloidin (red). (scale bars 100 µm). PanIN: arrowheads.

d H&E of the pancreas of control and *Ptf1a$^{Cre/+}$;Fra1$^{lox/lox}$* mice (scale bar 50 µm).

e H&E staining of three months old *Ptf1a$^{Cre/+}$;LSL-Kras$^{G12D/+}$* (*KC*) mice and *Ptf1a$^{Cre/+}$;LSL-Kras$^{G12D/+}$;Fra1$^{lox/lox}$* (*KCF*) mice (scale bar 50 µm).

f Quantification of ADMs and murine PanINs in 3 (left panel) and 6 (middle panel) months old *Ptf1a$^{Cre/+}$;LSL-Kras$^{G12D/+}$* (*KC*) mice and *Ptf1a$^{Cre/+}$;LSL-Kras$^{G12D/+}$;Fra1$^{lox/lox}$* (*KCF*) mice. Right panel: Quantification of low grade murine PanIN1A/1B and high grade murine PanIN2/3 in 6 months old *Ptf1a$^{Cre/+}$;LSL-Kras$^{G12D/+}$* (*KC*) mice and *Ptf1a$^{Cre/+}$;LSL-Kras$^{G12D/+}$;Fra1$^{lox/lox}$* (*KCF*) mice. * p value of a two tailed unpaired students t-test $p<0.05$.

g Kaplan-Meier curve of *Ptf1a$^{Cre/+}$;LSL-Kras$^{G12D/+}$;Fra1$^{lox/lox}$* (*KCF*) (n=9; median survival 489 days) mice.

h H&E staining of a PDAC of a *Ptf1a*$^{Cre/+}$*;LSL-Kras*$^{G12D/+}$*;Fra1*$^{lox/lox}$ (*KCF*) mouse (scale bar 200 µm).

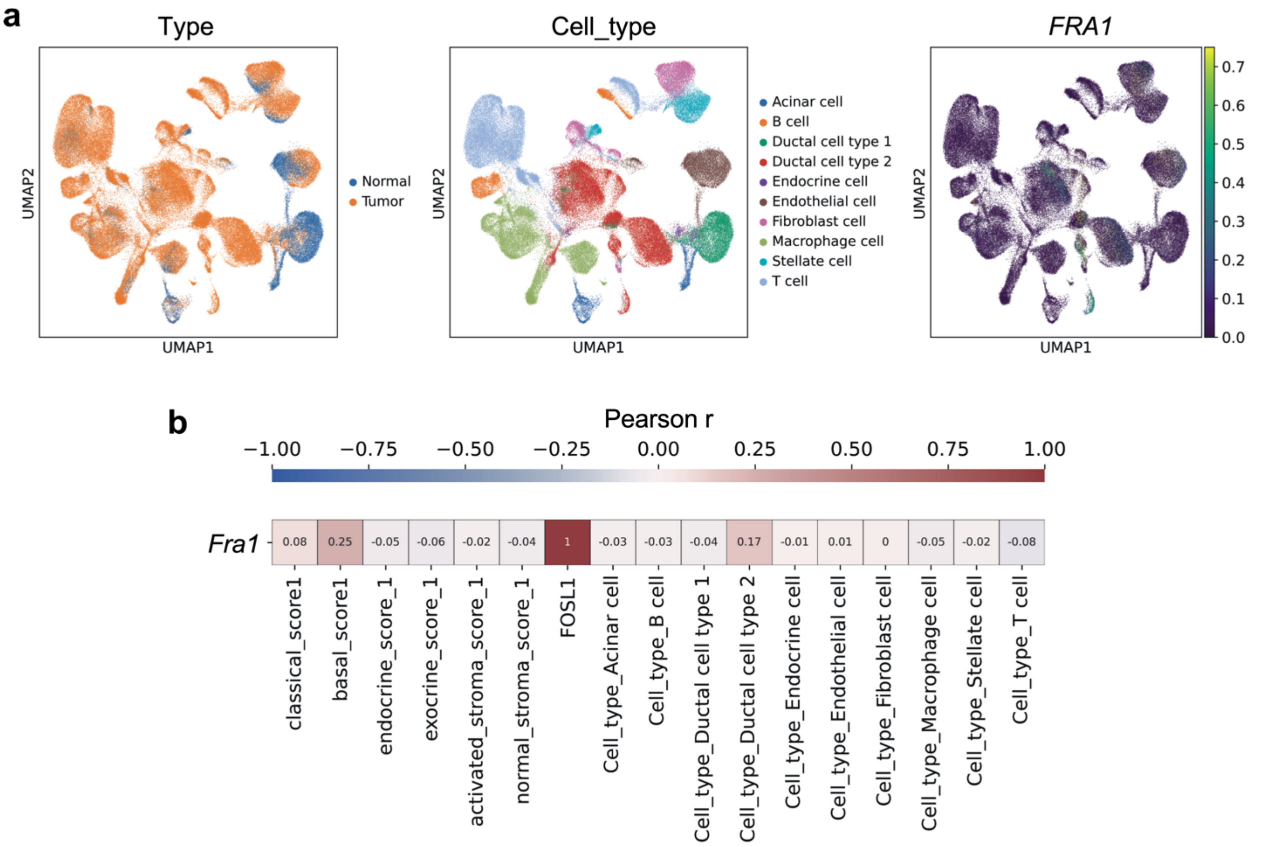

Figure. S3. Single-cell RNA-seq analysis of *FRA1* expression in human PDAC.

a UMAPs showing sample type (tumor versus normal) (left panel), cell type (middle panel) and *FRA1* expression from an integrated human PDAC single-cell RNA-seq dataset. Data are derived from a previously published reference single-cell atlas of PDAC from six datasets containing more than 70 samples and over 130,000 cells.

b FRA1 expression is connected to the basal-like subtype. Pearson correlation between *FRA1* mRNA expression and gene signature scores for the different cell types or tumor subtypes.

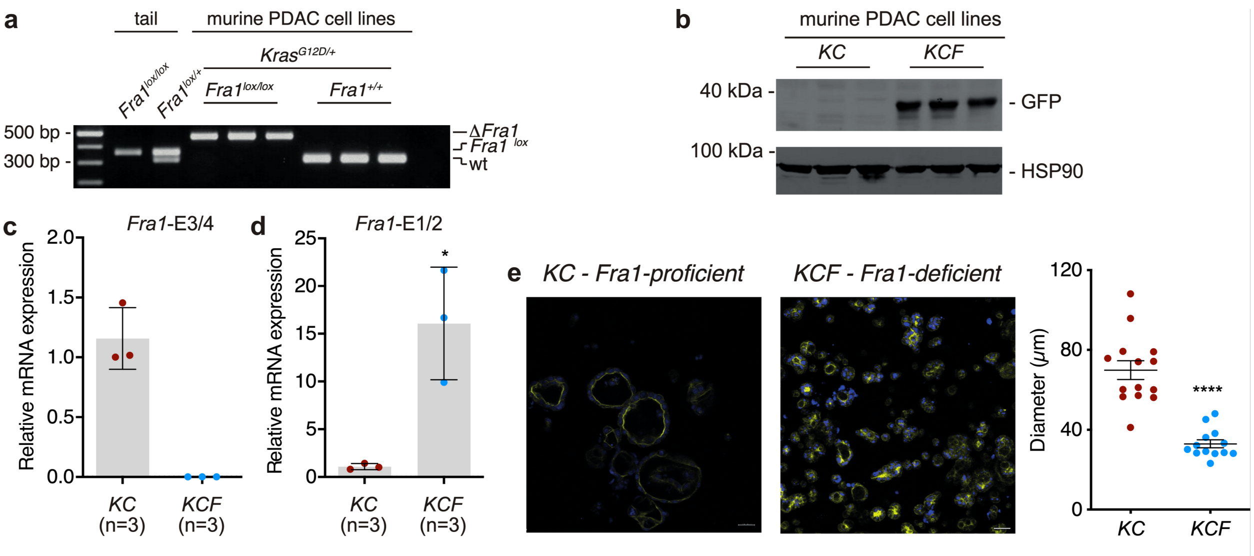

Figure. S4. *Fra1*-deficient murine PDAC cells.

a *Fra1* genotyping PCR in three *Ptf1a*$^{Cre/+}$;*LSL-Kras*$^{G12D/+}$ (*KC*) and three *Ptf1a*$^{Cre/+}$;*LSL-Kras*$^{G12D/+}$;*Fra1*$^{lox/lox}$ (*KCF*) PDAC cell lines. As controls, tail DNA from *Fra1*$^{lox/lox}$ and *Fra1*$^{lox/+}$ mice were used.

b GFP western blot in three *Ptf1a*$^{Cre/+}$;*LSL-Kras*$^{G12D/+}$ (*KC*) and three *Ptf1a*$^{Cre/+}$;*LSL-Kras*$^{G12D/+}$;*Fra1*$^{lox/lox}$ (*KCF*) PDAC cell lines. (HSP90: loading control). One lysate was analyzed.

c-d Relative *Fra1* mRNA expression in three *Ptf1a*$^{Cre/+}$;*LSL-Kras*$^{G12D/+}$ (*KC*) and three *Ptf1a*$^{Cre/+}$;*LSL-Kras*$^{G12D/+}$;*Fra1*$^{lox/lox}$ (*KCF*) PDAC cell lines was determined by qPCR using *cyclophilin A* mRNA expression as reference. For comparison, *Fra1* mRNA expression in one KC PDAC cell line was arbitrarily set to 1. C) Floxed Exon 3 and 4 specific qPCR. D)

Exon 1 and 2 specific qPCR. One mRNA preparation per cell line was analyzed in technical triplicates.

e A *Ptf1a*$^{Cre/+}$;*LSL-Kras*$^{G12D/+}$ (*KC*) (left panel) and a *Ptf1a*$^{Cre/+}$;*LSL-Kras*$^{G12D/+}$;*Fra1*$^{lox/lox}$ (*KCF*) (middle panel) PDAC cell line were cultured in a 3D matrix. Right panel: Quantification of diameter of the formed spheroids. **** unpaired t-test $p<0.0001$.

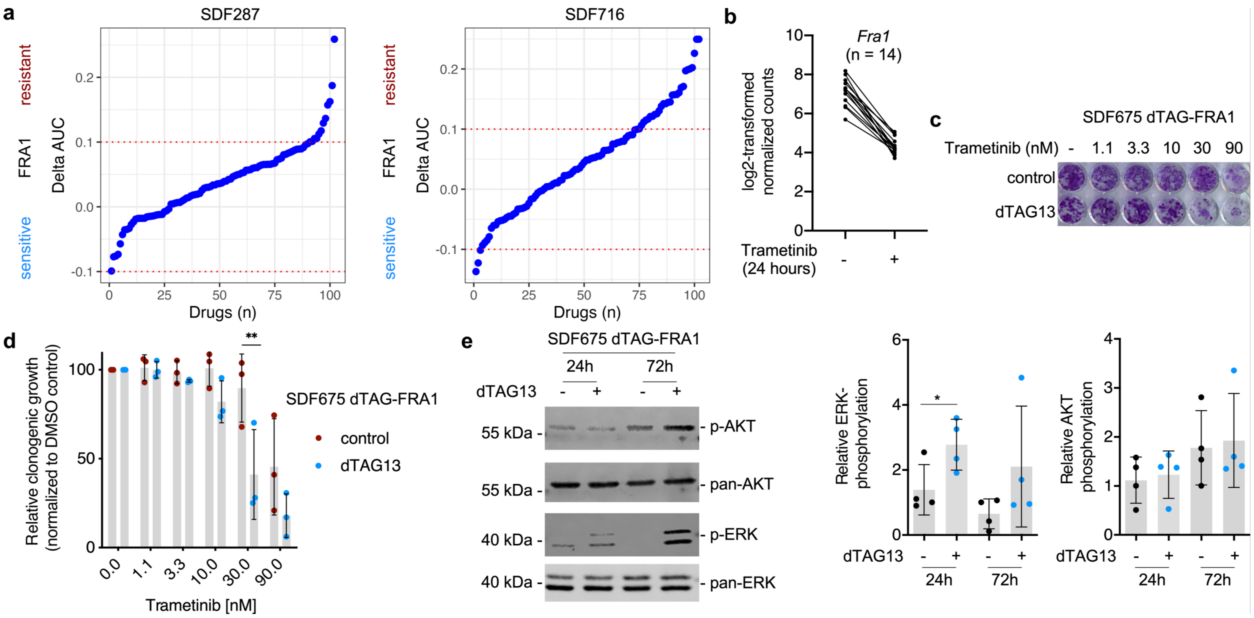

Figure. S5. Drug screening of isogenic *Fra1*-deficient and -proficient models.

a Distribution of delta-AUC values from the drug screening experiment in isogenic *Fra1*-proficient and -deficient cell lines. A drug screen with 102 compounds targeting various cancer-relevant pathways was performed in the *Ptf1a*$^{Cre/+}$*;LSL-Kras*$^{G12D/+}$*; Fra1*$^{lox/lox}$ (*KCF*) PDAC cell lines SDF287 and SDF716, which were transduced either with the pLenti-FRA1 construct for reconstitution of FRA1 expression or with pLenti-RFP reporter construct as control. Cells were treated in technical duplicates and cell viability was assessed by CellTiter-Glo (Promega) upon 72 hours of treatment. Area-under-the-Curve (AUC) values were determined based on the GRmetrics methodology. deltaAUC=AUC_{FRA}-AUC_{RFP}. Red-dotted line marks delta AUC +/- 0.1. Left panel: delta-AUC distribution for SDF287. Right panel: delta-AUC distribution for SDF716.

b *Fra1* mRNA expression is reduced after MEK inhibitor treatment. Displayed are the log2-transformed normalized counts of *Fra1* mRNA expression in 14 murine PDAC cell lines upon treatment with the MEK inhibitor Trametinib (10 nM) for 24 hours in comparison to untreated controls. Data can be accessed via EBIArrayExpress repository with accession number E-MTAB-11187.

c, d Perturbation of FRA1 sensitizes to MEK inhibition in clonogenic assays. The cell line SDF675 isolated from a murine PDAC with the genotype *Pdx1-Flp;FSF-Kras*$^{G12D/+}$, *p53*$^{frt/+}$; *Fra1*$^{lox/lox}$ was transduced with FKBP12^{F36V}-FRA1 (dTAG-FRA1) and pInducer-iCre vectors was pre-treated first for 8 days with doxycycline to induce complete recombination of the endogenous floxed *Fra1* alleles by the doxycycline-inducible iCre recombinase. Cells were then seeded in 24-well plates in medium containing 1 µM dTAG13 degrader (or equal volume of DMSO as vehicle control) and treated the following day with the indicated doses of the MEK1/2 inhibitor Trametinib (in technical duplicates). Cells were stained with crystal violet, when the cells in the control well reached near confluency around 7 days after seeding. One representative clonogenic assay from three independent experiments is displayed in C). D) Quantification of Clonogenic Growth from C). Crystal violet stainings were solubilized with 1% SDS and OD values measured. Displayed are the mean +/- SD from the relative OD values (normalized to DMSO treated wells) from three independent experiments (each experiment performed in technical duplicates). Each dot represents one independent experiment. P values from 2way ANOVA with multiple comparisons. ** $P \leq 0.01$

e Increased expression of phospho-ERK upon perturbation of FRA1. Left panel: Western Blot of phospho-ERK, total ERK (pan-ERK), phospho-AKT (Ser473) and total AKT (pan-AKT) upon perturbation of FRA1 in the SDF675 dTAG-FRA1 cell line by treatment for 24 and 72 hours with the small-molecule degrader dTAG13 (1 µM), which induces degradation of the dTAG-FRA1 fusion protein. Right panels: Quantification of phospho-ERK and phospho-AKT expression from the Western Blots. Shown is the relative expression of phosphorylated to total protein upon treatment with dTAG13 for 24 and 72 hours. Values are normalized to DMSO-treated controls. Each dot represents one independent experiment. Bars represent the mean +/- SD from four independent experiments. *P-value of an unpaired t-test <0.05.

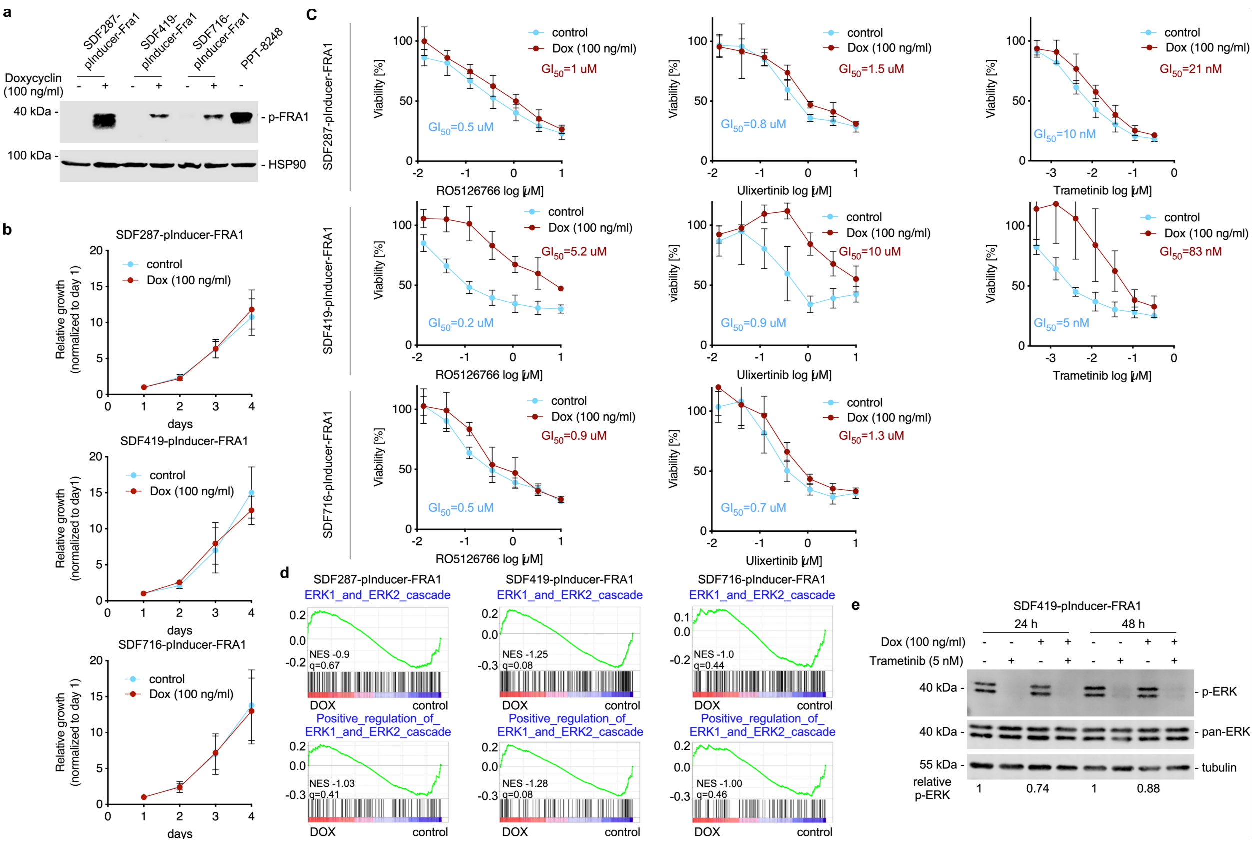

Figure. S6. Increased MAPK inhibitor resistance upon short-term doxycycline-inducible expression of FRA1

a Western Blot showing expression of phospho-FRA1 upon doxycycline treatment of KCF cell lines transduced with pInducer-FRA1. The three KCF cell lines SDF287, SDF419 and SDF716 transduced with pInducer-FRA1 were treated with 100 ng/mL doxycycline for 24 hours to induce expression of FRA1. The KC cell line PPT-8248 served as control. HSP90 was used as a loading control. One representative image out of two independent experiments is shown.

b Relative cell growth of the three KCF cell lines SDF287 (upper panel), SDF419 (middle panel) and SDF716 (lower panel) transduced with pInducer-FRA1 allowing for doxycycline-inducible expression of FRA1. For each condition, 1,000 cells were seeded

per well and treated on the following day with 100 ng/mL doxycycline or vehicle control. Relative growth was determined by MTT assays. The absorbance values of each cell line were determined on day 1, 2, 3 and 4 after seeding and are displayed as relative values normalized to day 1. Results are shown as mean +/- SD from three independent experiments.

c Increased resistance to MAPK inhibition upon short-term doxycycline-induced expression of FRA1. The KCF cell lines SDF287 (upper panel), SDF419 (middle panel) and SDF716 (lower panel) transduced with pInducer-FRA1 were seeded out in 96-well plates (1,000/well) in growth medium in the presence or absence of 100 ng/mL doxycycline. On the following day, the cells were treated in technical triplicates with 7-point serial dilutions of the dual RAF/MEK inhibitor RO5126766 (left panel), the ERK inhibitor Ulixertinib (middle) or the MEK inhibitor Trametinib (right panel, only SDF287 and SDF419 treated). Viability was determined by MTT assay after 72 hours of drug treatment and is displayed as relative viability normalized to vehicle-treated controls. Values represent the mean of three independent experiments +/- SD. GI_{50} values were calculated by non-linear regression and are indicated in the respective figures.

d ERK1/2 gene signatures upon doxycycline-induced FRA1 expression. RNA-seq with subsequent geneset enrichment analysis was performed in KCF cells SDF287 (left panel), SDF419 (middle panel) and SDF716 (right panel) transduced with pInducer-FRA1 and cultivated for 24 hours in the absence or presence of doxycycline (100 ng/mL). Gene sets from the Gene Ontology Biological Process (GOBP) database were used and enrichment

plots for “ERK1 and ERK2 cascade” (upper panel) and “Positive Regulation of ERK1 and ERK2 cascade” (lower panel) are shown. NES and q-Values are indicated.

e Western Blot of phospho-ERK expression in the SDF419 pInducer-FRA1 cell line upon treatment with doxycycline and Trametinib. SDF419 pInducer-FRA1 cells were treated with Trametinib for 24 and 48 hours in the presence or absence of doxycycline (100 ng/mL) and analyzed by Western Blot for expression of phospho- and pan-ERK. Ratios of phospho-ERK to pan-ERK expression are indicated. Tubulin served as loading control. Results are from n = 1 experiment.
